# Comparative transcriptome analysis of fiber and nonfiber tissues to identify the genes preferentially expressed in fiber development in *Gossypium hirsutum*

**DOI:** 10.1038/s41598-021-01829-8

**Published:** 2021-11-24

**Authors:** Jiangtao Yang, Lihua Gao, Xiaojing Liu, Xiaochun Zhang, Xujing Wang, Zhixing Wang

**Affiliations:** 1 Biotechnology Research Institute, MOA Key Laboratory on Safety Assessment (Molecular) of Agri-GMO, Chinese Academy of Agricultural Sciences, Beijing, 100081 China; 2 School of Life Sciences, Langfang Normal University, Langfang, 065000 China

**Keywords:** Biological techniques, Biotechnology, Molecular biology, Plant sciences

## Abstract

Cotton is an important natural fiber crop and economic crop worldwide. The quality of cotton fiber directly determines the quality of cotton textiles. Identifying cotton fiber development-related genes and exploring their biological functions will not only help to better understand the elongation and development mechanisms of cotton fibers but also provide a theoretical basis for the cultivation of new cotton varieties with excellent fiber quality. In this study, RNA sequencing technology was used to construct transcriptome databases for different nonfiber tissues (root, leaf, anther and stigma) and fiber developmental stages (7 days post-anthesis (DPA), 14 DPA, and 26 DPA) of upland cotton Coker 312. The sizes of the seven transcriptome databases constructed ranged from 4.43 to 5.20 Gb, corresponding to approximately twice the genome size of *Gossypium hirsutum* (2.5 Gb). Among the obtained clean reads, 83.32% to 88.22% could be compared to the upland cotton TM-1 reference genome. By analyzing the differential gene expression profiles of the transcriptome libraries of fiber and nonfiber tissues, we obtained 1205, 1135 and 937 genes with significantly upregulated expression at 7 DPA, 14 DPA and 26 DPA, respectively, and 124, 179 and 213 genes with significantly downregulated expression. Subsequently, Gene Ontology (GO) enrichment and Kyoto Encyclopedia of Genes and Genomes (KEGG) metabolic pathway analyses were performed, which revealed that these genes were mainly involved in catalytic activity, carbohydrate metabolism, the cell membrane and organelles, signal transduction and other functions and metabolic pathways. Through gene annotation analysis, many transcription factors and genes related to fiber development were screened. Thirty-six genes were randomly selected from the significantly upregulated genes in fiber, and expression profile analysis was performed using qRT-PCR. The results were highly consistent with the gene expression profile analyzed by RNA-seq, and all of the genes were specifically or predominantly expressed in fiber. Therefore, our RNA sequencing-based comparative transcriptome analysis will lay a foundation for future research to provide new genetic resources for the genetic engineering of improved cotton fiber quality and for cultivating new transgenic cotton germplasms for fiber quality improvement.

## Introduction

Cotton is not only an important economic crop worldwide but also a natural fiber and oil crop that can sustainably provide renewable resources and has a strong ability to survive in various environments. Upland cotton has strong environmental adaptability and high fiber production. It has been planted in large quantities worldwide, and its output accounts for approximately 95% of all planted cotton^1^. However, fiber quality, such as fiber length, uniformity index, micronaire value, breaking strength, and fiber elongation, is relatively ordinary. The elongation and development process of cotton fiber is a complex and orderly regulation process involving multiple genes and pathways. The yield and quality of cotton fiber are more sensitive to the external environment^2^. Because the quality of cotton fiber directly determines the quality of textiles, improving the yield and quality of cotton fiber has always been the focus of attention in the process of human cotton domestication. With the rapid development of science and technology and continuous scientific exploration, sequencing technology has gradually become a very widely used methodology in scientific research. With the development of mRNA sequencing technology, genome sequencing technology, resequencing technology and phenotype evaluation methods for cotton, important technologies and resources for studying the biological mechanisms of cotton fiber elongation have been developed^3^. Therefore. Breeders can use new biotechnology to develop new varieties of upland cotton with excellent fiber quality and high yield, which is of great benefit not only to upland cotton breeding but also to the global textile industry.

Recently, RNA deep sequencing technology has provided a platform for the analysis of differences in gene expression^4^. RNA-seq technology has been widely used in transcriptome studies of *Arabidopsis thaliana*^5^, *Populus trichocarpa*^6^, *Glycine max*^7^, *Oryza sativa*^8^, *Medicago sativa*^9^, *Gossypium hirsutum*^10^, *Brassica napus*^11^, *Triticum aestivuml*^12^, and *Zea mays*^13^, among others. The extensive application of RNA-seq technology has promoted the study of fiber elongation and development and provided strong technical support for the identification of genes with fiber-specific or fiber-dominant expression in cotton. In the study of cotton fiber, this technology is mainly applied for the investigation of differentially expressed genes (DEGs) between certain tissues of different cotton varieties^14^, between different tissues of the same cotton variety^15^, and during different developmental periods in the same cotton tissue^16^. By analyzing a transcriptome database, we can identify significant DEGs and then screen key genes that play important roles in different tissues or different developmental stages as candidate research objects for detailed functional analysis. For example, Padmalatha et al. sequenced the transcriptome of cotton fibers at different developmental stages under drought stress treatment and found that pectin modification and cytoskeletal protein-related genes play important roles in the initial differentiation stage of fiber primordial cells. These research results will help researchers develop drought-tolerant cotton cultivars without compromising fiber quality^17^. Using *Gossypium hirsutum* and *Gossypium barbadense* as materials, Li et al. sequenced the transcriptomes of fiber samples from different developmental stages (10 DPA, 15 DPA, 20 DPA, 25 DPA and 28 DPA) using Illumina HiSeq 2000 sequencing technology. A total of 1801 DEGs were identified, including 902 upregulated and 899 downregulated DEGs, which were mainly involved in the cell wall, cytoskeleton, transcription, and translation regulation. These findings lay a solid foundation for improving the fiber yield and quality^18^. Hu et al. performed transcriptome sequencing using 0 DPA and 5 DPA fibers from the Xu 142 cultivar and its mutant Xu *142fl*. A total of 2641 new genes, 35,802 long noncoding RNAs (lncRNAs), and 2262 circular RNAs were identified. Three lncRNAs were selected as research objects in studies involving virus-induced gene silencing (VIGS) technology. It was found that these lncRNAs play an important role in the development of cotton fiber elongation^19^. Parekh et al. performed transcriptome sequencing of *Gossypium herbaceum* fibers from different stages and obtained 20,125 unigenes. They predicted some transcription factors that play an important role in the development of cotton fiber elongation^15^. Xu et al. revealed the evolution of the reactive oxygen species (ROS) network and the regulation of fiber development in cotton. It was found that the ROS network-mediated signaling pathway enhanced the regulatory mechanisms of fiber elongation and development in cotton^20^.

Many studies have used RNA-seq technology to discover genes that are specifically or predominantly expressed in association with cotton fiber development. For example, Wan et al. searched for genes that were specifically expressed or regulated fiber elongation by sequencing the transcriptome of *Gossypium hirsutum* Xu 142 and its fuzzless mutant Xu *142fl*^21^. Man et al. used fibers from different developmental stages of *Gossypium hirsutum* and *Gossypium barbadense* as research objects and performed transcriptome sequencing analysis to screen for excellent genes determining fiber quality^22^. Li et al. searched for genes determining excellent fiber properties of cotton via transcriptome sequencing analysis of two inbred lines from a *Gossypium hirsutum* × *Gossypium barbadense* backcross^23^. However, when analyzing transcriptome sequencing data from Xu 142 and the Xu *142fl* mutant, many DEGs were found, which might be due to random mutations causing marked differential expression of many genes that were not related to fiber development. This occurrence makes it difficult to screen genes for superior fiber traits. In addition, analysis of transcriptome sequencing data from different developmental stages of *Gossypium hirsutum* and *Gossypium barbadense* revealed that many genes with significant expression differences could be screened due to the very different genetic backgrounds of these species. However, these DEGs may result from different genetic backgrounds rather than being associated with good fiber quality and yield traits. Therefore, it is difficult to select excellent fiber quality genes by comparing the expression of fiber quality- and yield-related genes between *Gossypium hirsutum* and *Gossypium barbadense*.

In this study, transcriptome sequencing was performed on different tissues of *Gossypium hirsutum* Coker 312, and the DEGs were compared between fiber and nonfiber tissues to effectively narrow the selection range of candidate genes related to good quality traits in fiber development. Thus, information on genes that directly participate in fiber synthesis or regulate fiber development during fiber elongation and development could be obtained quickly. This study provides important genetic resources for breeding new cotton germplasms with excellent fiber quality.

## Results

### Detection of total RNA quality in different cotton tissues

**Figure 1:**
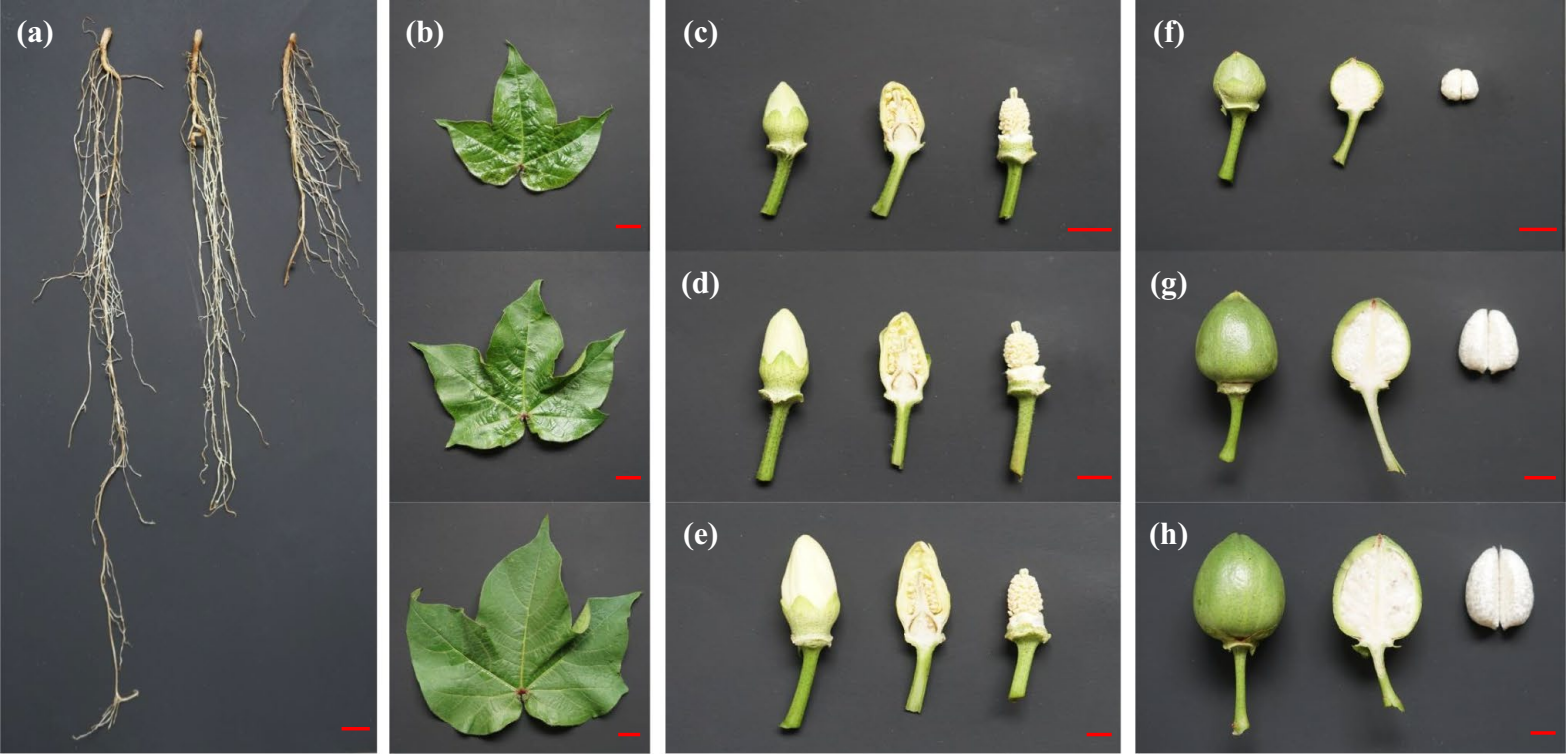
Seven different tissues of upland cotton Coker 312 were sampled. (**a**, **b**): Samples of roots and leaves were collected at 15, 25 and 35 days after germination; (**c**–**e**): samples of anthers and stigmas were collected at − 3, − 2 and − 1 days before anthesis; (**f**–**h**): samples of fibers were collected at 7, 14 and 26 DPA. The length of the red ruler line represents the actual measured length of 1 cm.

Seven different tissues of upland cotton Coker 312 were sampled (Fig. 1). Samples of roots and leaves were collected at 15, 25 and 35 days after germination (Fig. 1a,b). Samples of anthers and stigmas were collected at − 3, − 2 and − 1 days before anthesis (Fig. 1c–e). Samples of fibers were collected at 7, 14 and 26 DPA (Fig. 1f–h). Detection of total RNA by agarose gel electrophoresis showed that the RNA from seven tissues exhibited two complete rRNA bands and that the 28S rRNA band was approximately twice as bright as the 18S rRNA band. The results indicated that the integrity of these total RNAs was relatively complete, and no degradation was observed (Fig. S1). The concentration and purity of the total RNA were measured with a Nanodrop 2000 spectrophotometer. The concentration of each sample was higher than 150 ng/μL, the OD260/280 was approximately 1.9, and the OD260/230 was greater than 2.0. Therefore, the RNA concentration and purity of these samples were relatively high, reaching the standard for A-level library construction (Table S1). The next step of database building and sequencing was then conducted.

### Transcriptome sequencing and comparison with the reference genome

On the Illumina HiSeq™ 2000 platform, high-throughput RNA sequencing generated 343 million raw reads from seven cotton tissues, with more than 46.59 million reads per tissue. A total of 332 million clean reads (96.79%) were obtained from the total raw reads after discarding adapters, low-quality reads and raw reads filtered according to an N greater than 5%. Two biological replicates were evaluated for each line, and the results indicated that the RNA-seq data were in good agreement (0.936 < R2 < 0.982). For all sequence data, the average Q20, Q30, and GC contents were 98.88%, 93.39% and 43.86%, respectively, and the error rates for all samples ranged from 0.01 to 0.02%. Overall, 83.33%-88.22% of the high-quality clean reads were mapped to the reference genome of *Gossypium hirsutum* TM-1 using TopHat29 (Table 1). The distribution of the numbers of genes expressed in each tissue sample (Table S2) and the sequencing coverage of the gene transcripts (Fig. S2) were analyzed. There were 3774–4481 genes expressed in high abundance. Among the mapped reads, 78.75–88.03% were distributed in exon regions, 1.54–7.81% were located in introns, and 10.09–17.26% were located in intergenic regions. Three types of mapping data were obtained: (1) multiple mapping (21–25.66%) and unique mapping reads (60.24–65.4%), (2) forward mapping (36.84–39.38%) and reverse mapping reads (36.75–39.22%), and (3) nonspliced reads (56.68–61.93%) and spliced reads (13.23–21.65%) (Table 1).

**Table 1:** Statistical table of transcriptome sequencing data.

Samples	Root	Leaf	Anther	Stigma	Fiber_7	Fiber_14	Fiber_26
Raw reads number	47,620,958	49,175,082	53,347,998	52,705,146	46,738,588	46,747,314	46,593,006
Clean read number	46,795,956	46,682,610	52,033,422	51,138,576	44,381,584	45,378,512	45,477,042
Clean read rate (%)	98.28	94.93	97.54	97.03	94.96	97.07	97.6
Adapter polluted read rate (%)	0.5	1.14	0.45	0.43	0.45	0.38	0.45
Ns read rate (%)	0	0.03	0.02	0	0.02	0.01	0.01
Low-quality read rate (%)	1.22	3.93	1.99	2.54	4.57	2.54	1.95
Q20 bases rate (%)	99.19	99.07	98.71	98.93	98.57	98.58	99.08
Q30 bases rate (%)	94.69	94.68	92.45	93.42	92.01	91.96	94.50
GC content (%)	44	43	44	43	44	44	45
Mapped reads	41,205,838	41,184,069	45,664,386	42,609,136	39,058,763	39,856,938	39,064,126
Mapping rate (%)	88.05	88.22	87.76	83.32	88.01	87.83	85.9
Exon (%)	86.47	80.56	85.12	78.75	88.03	87.58	87.17
Intron (%)	2.46	2.19	2.41	7.81	1.88	1.54	1.58
Intergenic (%)	11.07	17.26	12.47	13.44	10.09	10.88	11.24
Uniq map rate (%)	64.59	63.68	62.79	62.32	65.40	64.53	60.24
Multi map rate (%)	23.47	24.54	24.97	21.00	22.61	23.30	25.66
Map to sense strand rate (%)	39.38	38.93	37.47	36.84	39.26	38.92	38.02
Map to antisense strand rate (%)	39.22	38.76	37.47	36.75	39.08	38.83	37.79
Nonsplice read rate (%)	59.29	61.93	56.87	60.36	56.68	57.32	58.29
Splice read rate (%)	19.32	15.76	18.07	13.23	21.65	20.43	17.52

A total of 76,772 genes were identified, and no fewer than 52,565 genes were expressed in the seven libraries. To test the correlations among the experimental samples, the expression of the genes in these seven libraries was analyzed using the Pearson correlation coefficient (PCC) (Fig. S3a). The results showed that 7 DPA fibers presented the greatest similarity to 14 DPA fibers (similarity as high as 0.732), followed by 26 DPA fibers (0.605), while the similarity to nonfiber tissues (root, leaf, anther and stigma) was lower. Therefore, gene expression was most similar in fibers from different elongation periods (7, 14 and 26 DPA); however, the gene expression between different tissues showed significant changes.

To further confirm the relationships among these seven different tissue libraries, principal component analysis (PCA) was performed on the expressed genes (Fig. S3b). The results showed that the expression of genes differed mainly among different tissues, except for the genes that were expressed in different periods in the fiber tissue. In the PCA, the gene expression pattern was different between different tissues, which was conducive to screening for cotton fiber superiority or specifically expressed genes and to some extent indirectly verified the reliability of our RNA-seq data.

### Type and abundance of alternative transcript splicing

**Figure 2:**
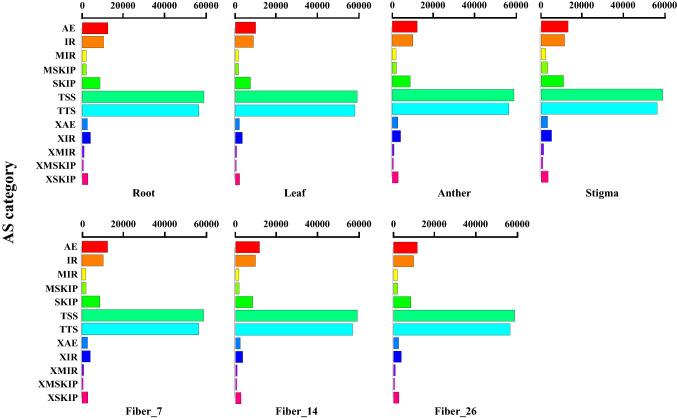
Types and numbers of variable splicing statistics. *AE* Alternative exon ends, *IR* intron retention, *MIR* multi-IR, *MSKIP* multiexon SKIP, *SKIP* skipped exon, *TSS* alternative 5′ first exon, *TTS* alternative 3′ last exon, *XAE* approximate AE, *XIR* approximate IR, *XMIR* approximate MIR, *XMSKIP* approximate MSKIP, *XSKIP* approximate SKIP.

Many genes can produce multiple mRNA transcripts via alternative splicing, and different mRNAs can be translated into different proteins. Thus, a gene can produce multiple proteins through alternative splicing, thereby greatly increasing the diversity of proteins. The alternative splicing events of transcripts predicted by StringTie (v1.0.4, http://ccb.jhu.edu/software/stringtie/) using ASprofile software (http://ccb.jhu.edu/software/ ASprofile/ index.shtml) were classified and counted as shown in Fig. 2. Many alternative splicing events were present in the seven different tissue samples, and the results showed that the overall pattern of alternative splicing events was largely similar across all samples, with more than 79% being concentrated in the alternative 3′ last exon (TTS), alternative 5′ first exon (TSS), and alternative exon end (AE) categories. The results showed no difference between samples from different tissues of cotton, indicating that alternative splicing events proceeded steadily in different tissues or in different periods in the same tissue.

### Screening of DEGs and overall transcriptome sequencing analysis

**Figure 3:**
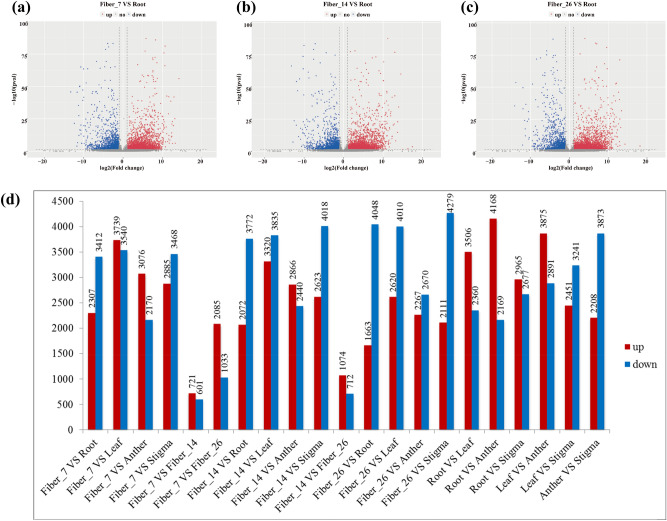
Upregulation and downregulation of DEGs between different tissues. (**a**–**c**) Volcano plot of DEGs (red dots represent upregulated DEGs, blue dots represent downregulated DEGs, gray dots represent genes that are not differentially expressed, the abscissa represents the fold change in gene expression in different samples, and the ordinate represents statistically significant differences in gene expression). (**d**) Comparison of the upregulation and downregulation of DEGs between each pair of samples in different tissues (red columns represent upregulated DEGs, blue columns represent downregulated DEGs, the abscissa represents the name of the sample comparison group, and the ordinate represents the number of DEGs).

To screen DEGs in different tissues of cotton, we used the fragments per kilobase of transcript per million fragments (FPKM) method to measure the gene expression levels. According to the different FPKM values of the expressed genes in each tissue, the screening parameters for DEGs were set as follows: p value < 0.05 and |Log2(Fold Change)| ≥ 2. If the |Log2Fold Change)| value was larger, it meant that the expression difference of the gene between samples was higher, that is, high-abundance expression. According to the FPKM value of each gene in different tissues, cluster analysis and differential gene expression profiling were performed. From our data, a volcano plot and a histogram showing detailed information about the number of DEGs in each pairwise comparison were generated (Fig. 3). According to the sequencing results, volcano plots were drawn and screened according to the significant difference criteria (difference in gene expression > 2 and FDR ≤ 0.05), and statistically significant differences in gene expression were measured. The volcano plots showed that many genes were upregulated and downregulated in the pairwise comparisons (Fig. 3a–c). Histograms were generated to summarize the significant DEGs identified in the pairwise comparisons of all samples (Fig. 3d).

Subsequently, the transcriptome data from the pairwise comparisons were compared with gene annotation databases; 889–5017 genes showed matches in the Gene Ontology (GO) database, and 765–4329 genes showed matches in the Kyoto Encyclopedia of Genes and Genomes (KEGG) database (Table 2).

**Table 2:** Summary of annotated genes in each database for the pairwise comparisons.

Pairwise comparisons	GO	KEGG
Fiber_7 vs. root	4111	3550
Fiber_7 vs. leaf	5017	4329
Fiber_7 vs. anther	3640	3090
Fiber_7 vs. stigma	4397	3773
Fiber_7 vs. fiber_14	889	765
Fiber_7 vs. fiber_26	2135	1877
Fiber_14 vs. root	4208	3545
Fiber_14 vs. leaf	4897	4157
Fiber_14 vs. anther	3655	3047
Fiber_14 vs. stigma	4536	3841
Fiber_14 vs. fiber_26	1209	1028
Fiber_26 vs. root	4077	3443
Fiber_26 vs. leaf	4487	3795
Fiber_26 vs. anther	3364	2793
Fiber_26 vs. stigma	4372	3700
Root vs. leaf	4104	3507
Root vs. anther	4508	3849
Root vs. stigma	3902	3342
Leaf vs. anther	4594	3921
Leaf vs. stigma	3833	3378
Anther vs. stigma	4150	3514

### Screening of DEGs from Venn diagrams between fiber and nonfiber tissues at different developmental stages

**Figure 4:**
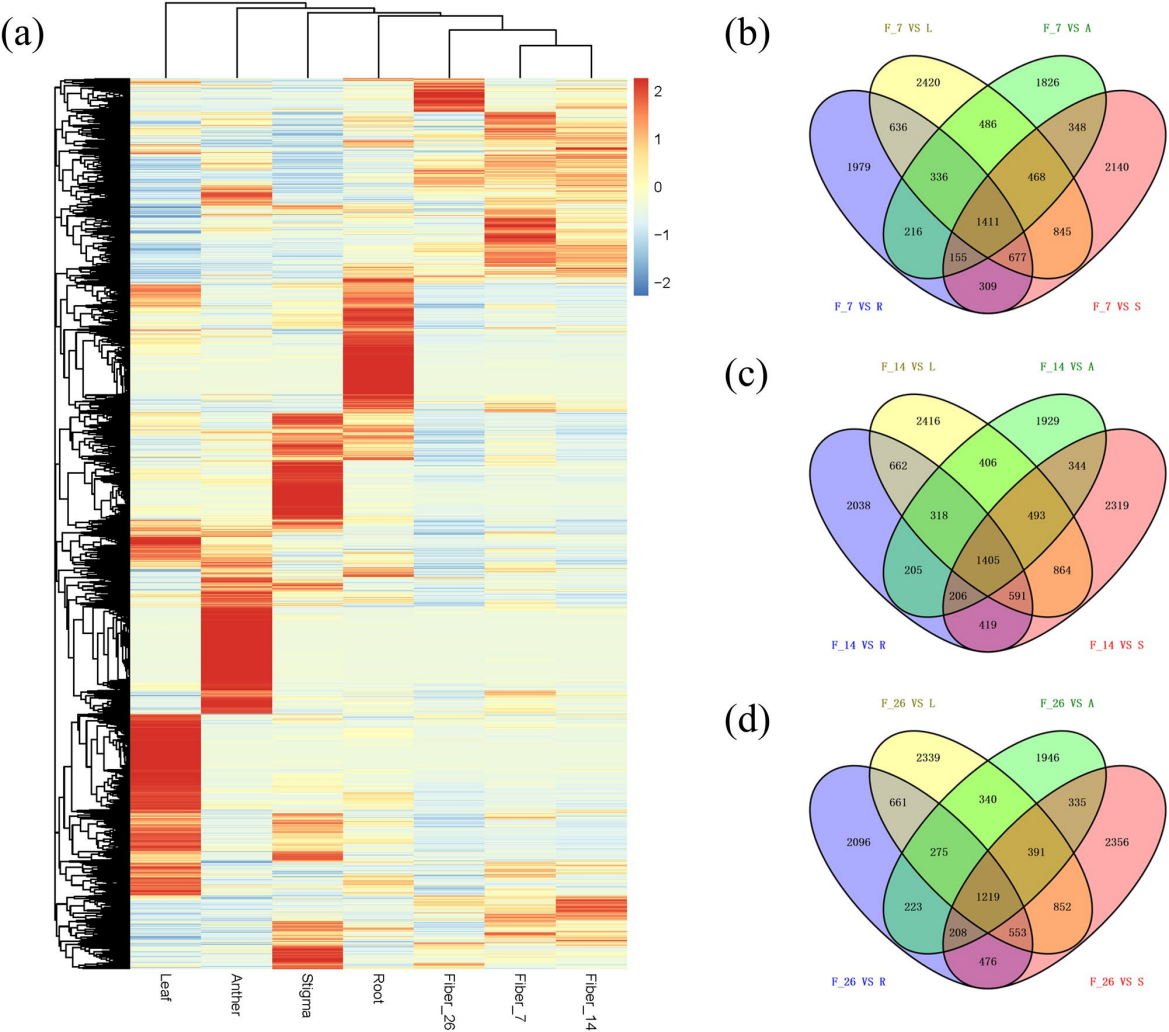
DEGs identified in seven different tissues from comparisons between fiber and nonfiber tissues. (**a**) Heatmap of DEGs (p value < 0.05, |Log2(Fold Change)| ≥ 2 at one sampling point); (**b**): Venn diagram analysis of the DEGs in 7 DPA fiber and nonfiber tissues; (**c**): Venn diagram analysis of the DEGs in 14 DPA fiber and nonfiber tissues; (**d**): Venn diagram analysis of the DEGs in 26 DPA fiber and nonfiber tissues. *A* anther, *L* leaf, *R* root, *S* stigma, *F_7* 7 DPA fiber, *F_14* 14 DPA fiber, *F_26* 26 DPA fiber.

To identify genes that were specifically or predominantly expressed during the period of fiber development and elongation, transcriptome data were used to generate heatmaps of DEGs, and Venn diagrams showing detailed information about the numbers of DEGs in each pairwise comparison were produced (Fig. 4). Overall, 7 DPA and 14 DPA fibers showed high similarity in their gene expression profiles, but they presented less similarity to the 26 DPA fibers; however, the similarity between the fiber profiles was higher than that between the nonfiber tissues (Fig. 4a). These results were consistent with the results of the PCC. Comparative analysis of DEGs between fiber and nonfiber tissues revealed 1411 DEGs in 7 DPA fibers (Fig. 4b), 1405 DEGs in 14 DPA fibers (Fig. 4c) and 1219 DEGs in 26 DPA fibers (Fig. 4d). Among the DEGs, there were 1205, 1135 and 937 upregulated DEGs (Fig. S4a–c) and 124, 179 and 213 downregulated DEGs (Fig. S4d–f) in 7 DPA, 14 DPA and 26 DPA fibers, respectively. The dynamic changes in the DEGs identified from comparative transcriptome analysis between fiber and nonfiber samples might reveal the regulatory mechanisms of key genes in fiber elongation development and quality formation.

### Functional annotations of DEGs in fiber and nonfiber transcriptome libraries

To identify genes related to fiber development and elongation, we analyzed DEGs from transcriptome data between fiber and nonfiber (root, leaf, anther and stigma) tissues. Compared with the transcriptome libraries of nonfiber tissues, 1411, 1405 and 1219 DEGs were identified in 7, 14 and 26 DPA fibers (Fig. 4b–d), respectively. Functional annotation analysis of the DEGs was performed according to GO terms and KEGG metabolic pathways.

**Figure 5:**
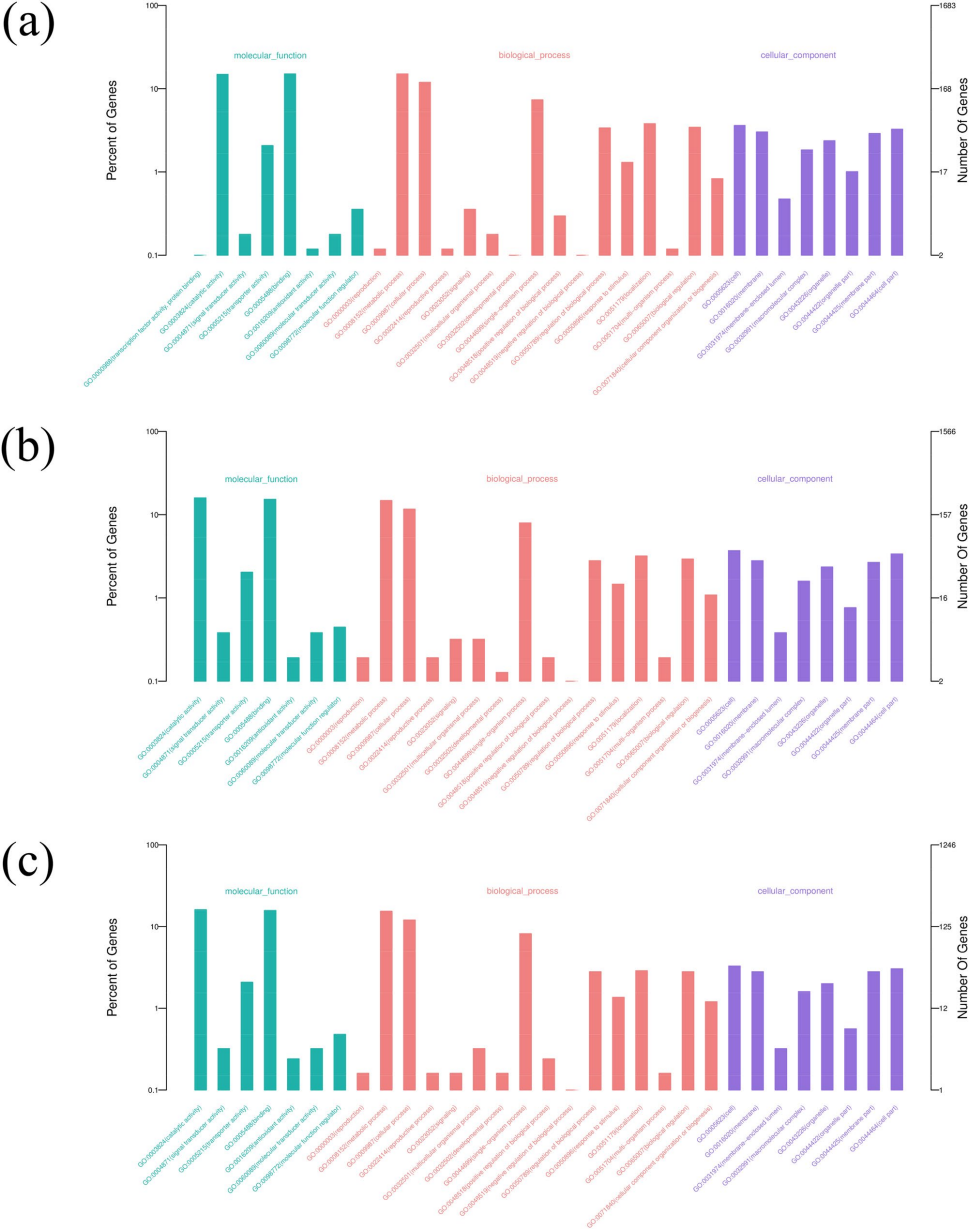
Functional annotation analysis of DEGs was conducted according to GO terms. (**a**) Enriched GO terms in 7 DPA fiber vs. nonfiber tissues; (**b**) enriched GO terms in 14 DPA fiber vs. nonfiber tissues; (**c**) enriched GO terms in 26 DPA fiber vs. nonfiber tissues.

In the GO term analysis, 576, 556 and 451 DEGs with functional annotations were enriched in 32, 31 and 31 GO groups in 7, 14 and 26 DPA fibers (Fig. 5a–c), respectively. During fiber elongation and development, among biological processes, the DEGs were significantly enriched in metabolic processes, cellular processes, single organism processes, and biological regulation processes. In the cell component category, the DEGs were mainly distributed in cells, membranes, and organelles. Among molecular functions, the DEGs were mainly enriched in functional groups, such as catalytic activity, binding, transport activity, and molecular function regulation. These results showed that the above functional groups could play an important role in fiber development and elongation.

**Figure 6:**
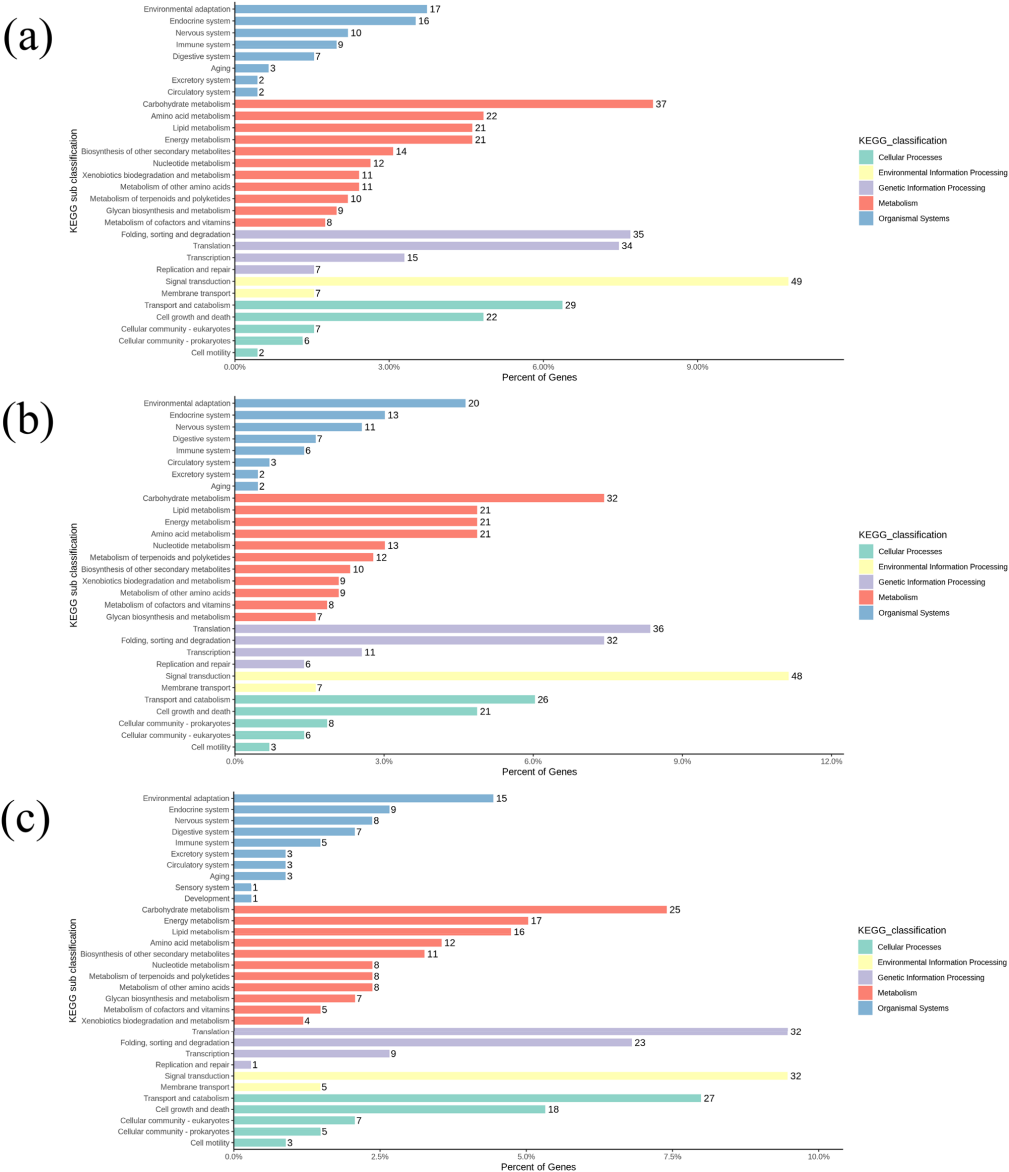
Functional annotation analysis of DEGs was carried out through KEGG metabolic pathway analysis. (**a**) Enriched KEGG metabolic pathways in 7 DPA fiber vs. nonfiber tissues; (**b**) enriched KEGG metabolic pathways in 14 DPA fiber vs. nonfiber tissues; (**c**) enriched KEGG metabolic pathways in 26 DPA fiber vs. nonfiber tissues.

In the KEGG metabolic pathway analysis, 455, 431 and 333 DEGs were significantly clustered into 30, 30 and 33 metabolic pathways in 7, 14 and 26 DPA fibers (Fig. 6a–c), respectively, which mainly included metabolic pathways such as those involved in signal transduction, carbohydrate metabolism, protein translation and processing, transportation and catabolism. These results showed that the above functional groups and metabolic pathways could play an important role in the stage of fiber development and elongation.

### Analysis of related functional genes during fiber elongation and development

**Figure 7:**
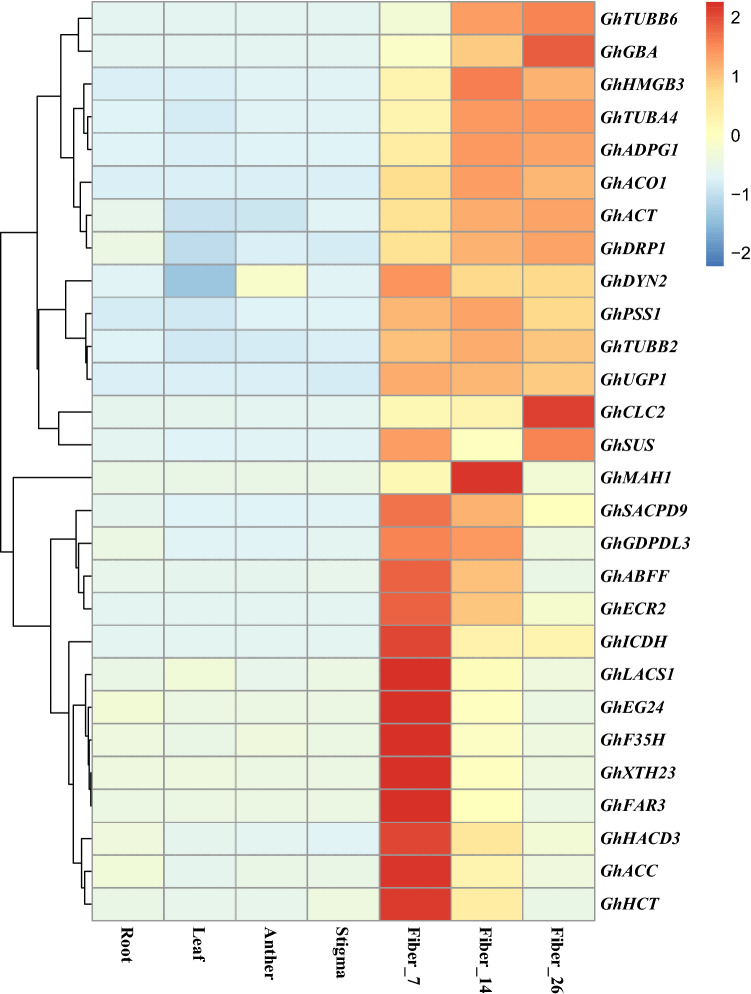
Heatmap of related functional genes during fiber elongation and development (p value < 0.05, |Log2(Fold Change)| ≥ 2 at one sampling point).

Fiber cell metabolism is very active during the fiber development and elongation stage, which is mainly manifested in the overexpression of three major groups of genes: (1) cytoskeleton-related genes; (2) cell wall structure and cellulose biosynthesis-related genes; and (3) energy and carbohydrate metabolism-related genes. A number of related functional genes that were differentially expressed during fiber elongation and development were selectively analyzed. The expression patterns of genes associated with fiber elongation development are shown in Fig. 7 and Table S3. Many upregulated genes were associated with cytoskeletal components, such as actin (*GhACT*), tubulin (*GhTUBA4*, *GhTUBB2*, *GhTUBB6*), kinesin (*GhPSS1*), clathrin (*GhCLC2*), and dynamin-related proteins *GhDRP1*), and these genes were predominantly or specifically expressed in cotton fiber tissues. The metabolic activities of various carbohydrate substances promote the rapid elongation of fibers, involving enzymes such as cellulose synthase (*GhCESA5*), sucrose synthase (*GhSUS*), glucosidase (*GhGBA*), phosphate uridylyltransferase (*GhUGP1*), polygalacturonase (*GhADPG1*) and other carbohydrate synthase or modification enzymes. Compared with nonfiber tissues, these genes were significantly upregulated at different fiber elongation stages. We also identified a number of genes involved in fatty acid metabolism and secondary metabolic processes, such as ultralong-chain enoyl coenzyme A reductase (*GhECR2*), fatty acyl coenzyme A reductase (*GhFAR3*), long-chain acyl coenzyme A synthase (*GhLACS1*), ultralong-chain-3-hydroxyacyl coenzyme A dehydratase (*GhHACD3*), alkane hydroxylase (*GhMAH1*), and phenylpropane compounds (*GhACO1*). In particular, most of the fatty acid metabolism-related genes were significantly upregulated in the early stage of fiber elongation (7 DPA), indicating that these genes play important roles in the early stage of cotton fiber elongation.

### Analysis of transcription factors during fiber elongation and development

**Figure 8:**
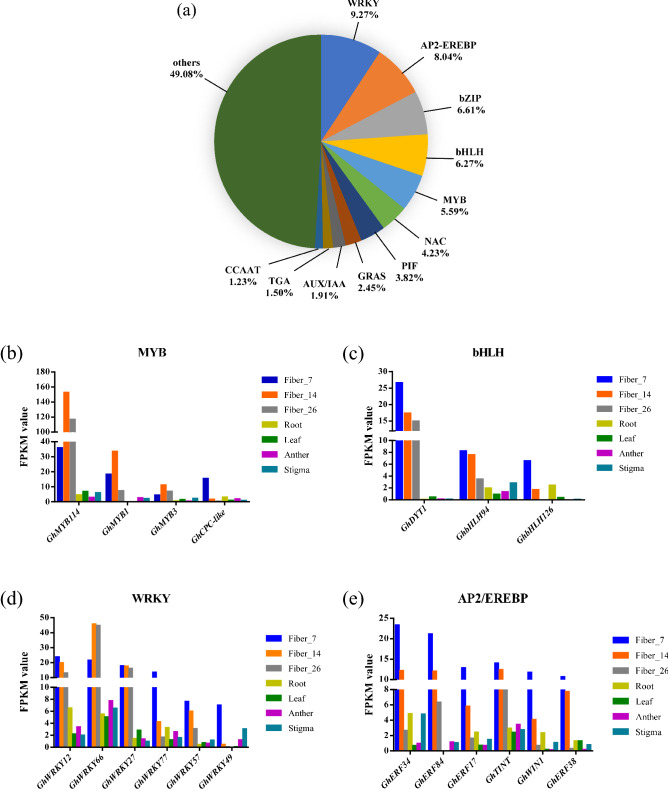
Statistics of transcription factor expression in different tissues of cotton. (**a**) Quantity and classification of transcription factor families. A total of 1467 different transcription factors were annotated in 46 transcription factor families. The numbers represent the percentages of transcription factor genes. (**b**–**e**) Detailed illustration of the expression of transcription factors related to fiber elongation and development. The x-axis indicates the distribution of transcription factors in seven different tissues of cotton. The y-axis represents the FPKM value of each transcription factor in different tissues.

Transcription factors can recognize and bind DNA sequences in a sequence-specific manner to regulate gene expression, forming a complex system that guides genome expression. Based on the criterion of |Log2(Fold Change)| ≥ 2 at one sampling point, a total of 1467 transcription factors from 46 transcription factor families were annotated between fiber and nonfiber tissues, including WRKY (136), AP2-EREBP (118), bZIP (97), bHLH (92), MYB (82), NAC (62), PIF (56), GRAS (36), AUX/IAA (28), TGA (22), and CCAAT (18) transcription factors (Fig. 8a). Among these transcription factors, 148 were significantly upregulated in fibers. Putative homologs of genes related to fiber development were identified in seven different cotton tissues (Fig. 8b–e). *GhMYB114*, *GhMYB1*, *GhMYB3* and *GhCPC-like* belong to the MYB transcription factor family. Among these genes, *GhMYB114*, *GhMYB1* and *GhMYB3* presented similar expression patterns and were significantly upregulated in 14 DPA fibers, with lower expression in the other fiber periods, while their expression levels in nonfiber tissues were very low. *GhCPC-like* was significantly upregulated in 7 DPA fibers. PTI-6 belongs to the AP2/EREBP transcription factor family, and three PTI-6-homologous genes (*GhERF34*, *GhERF38* and *GhERF84*) showed the same expression pattern and were upregulated in fiber tissue. However, their expression was significantly upregulated in the early stage of fiber elongation, and the expression level gradually decreased with the passage of developmental time. Two other AP2/EREBP transcription factors, RAP2 (*GhTINT*) and ERF (*GhWIN1*) also presented this expression pattern. Similarly, in the bHLH, bZIP and WRKY families, many transcription factors were found to be upregulated in fibers but showed gradually decreased expression with fiber development and were expressed at low levels or not at all in nonfiber tissues.

### Analysis of dominant or specific expression genes during fiber elongation and development

**Figure 9:**
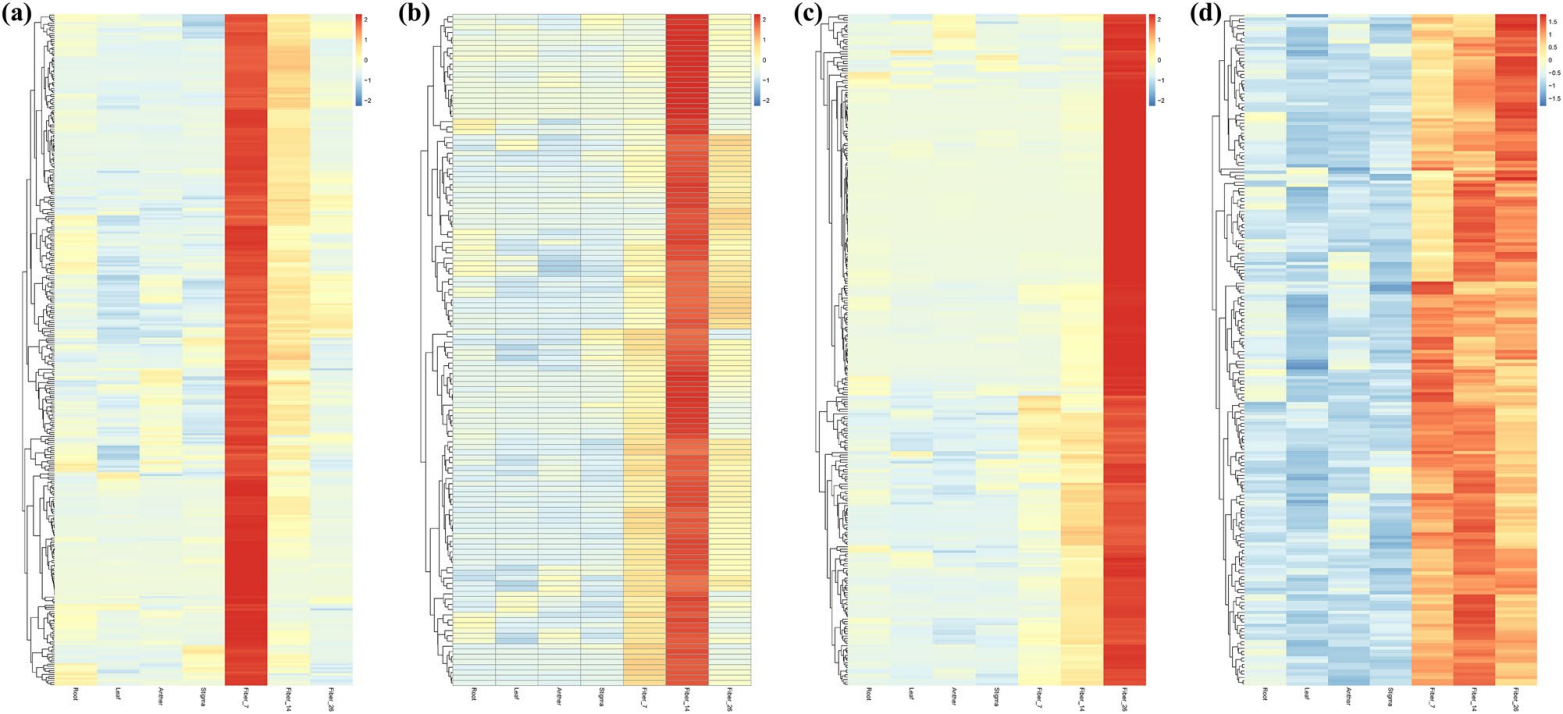
Heatmap of highly abundant genes expressed in different periods of fiber elongation and development (based on a p value < 0.05, the screening parameters between fibers and nonfibers were |Log2(Fold Change)| > 2, and the screening parameters between fibers at different elongation and development stages were 0.5 < |Log2(Fold Change)| < 1.5.). (**a**) Heatmap of highly abundant genes expressed in 7 DPA fibers; (**b**) heatmap of highly abundant genes expressed in 14 DPA fibers; (**c**) heatmap of highly abundant genes expressed in 26 DPA fibers; (**d**) heatmap of highly abundant genes expressed in the whole period of fiber elongation and development.

A large number of genes were expressed in different developmental stages and participated in the regulation of fiber cell elongation and development. In this study, some functional genes specifically or preferentially expressed in the process of fiber elongation and development were analyzed. Based on the p value ≤ 0.05, the screening parameters between fibers and nonfibers were |Log2(Fold Change)| ≥ 2, and the screening parameters between fibers at different elongation and development stages were 0.5 < |Log2(Fold Change)| < 1.5. Corresponding parameters were set to screen fiber-dominant genes. The expression pattern of dominant expression genes in fiber elongation development is shown in Table S4 and Fig. 9. A total of 330, 128 and 278 highly expressed genes were screened in Fiber_7, Fiber_14 and Fiber_26, respectively; among them, 154 (46.67%), 43 (33.60%) and 96 (34.53%) had clear gene annotations, and the rest were genes with unknown function. Simultaneously, 206 genes with high-abundance expression in the whole elongation and development period of fiber were also screened, of which 114 (55.34%) had clear gene annotations. Therefore, there were a large number of fiber-dominant or fiber-specifically expressed genes in the process of fiber elongation and development, which has not been reported previously.

### Validation of RNA-seq data by qRT-PCR

**Figure 10:**
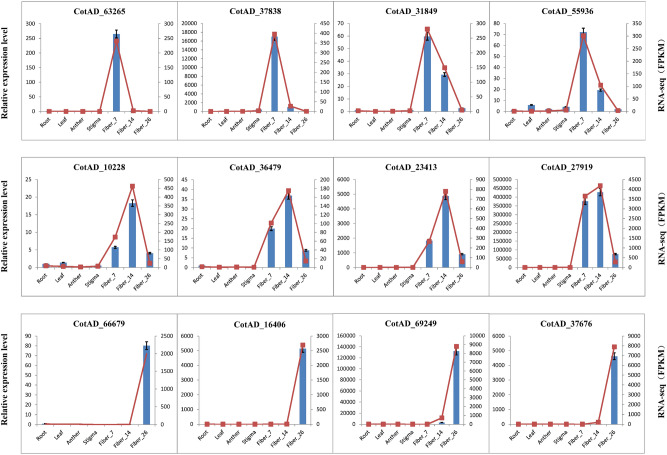
Validation of RNA-seq data by qRT-PCR. *Root* root, *Leaf* leaf, *Anther* anther, *Stigma* stigma, *Fiber_7* 7 DPA fiber, *Fiber_14* 14 DPA fiber, *Fiber_26* 26 DPA fiber; columns represent the Zresults of qRT-PCR, and zigzag lines represent the results of transcriptome sequencing.

To further validate the reliability of the RNA-seq results and the accuracy of the DEGs, twelve upregulated DEGs were randomly selected from each of the 7 DPA, 14 DPA and 26 DPA fiber transcriptome libraries, for a total of 36 genes. qRT-PCR was used to analyze the differential expression of genes between different tissues of cotton. Cotton Sad1 (NCBI Reference Sequence: NM_001327106.1) was used for relative gene expression normalization^24^. The qRT-PCR results showed that the 36 upregulated genes were all expressed specifically or predominantly in fibers (Fig. 10 and Fig. S5), among which 19 were specifically or preferentially expressed in 7 DPA fibers (e.g., CotAD_98043, CotAD_14327, CotAD_51137). Six upregulated genes were specifically or predominantly expressed in 14 DPA fibers, including CotAD_46959, CotAD_27919, CotAD_22244, CotAD_23413, CotAD_10228 and CotAD_36479. The remaining 11 upregulated genes were expressed specifically or preferentially in 26 DPA fibers. Comparative analysis of the qRT-PCR results and RNA-seq data revealed slight differences, but the expression trends of DEGs in different tissues were highly similar between the two groups of data. This result showed that the identification of genes that were specifically or predominantly expressed in fiber by comparing DEGs between different tissues resulted in improved accuracy. In conclusion, the transcriptome database of different cotton tissues constructed in this study presented high reliability.

## Discussion

### Transcriptome analysis of fiber and nonfiber tissues in cotton

Cotton fiber is an ideal model for studying cell elongation and cell wall construction in plants. Cotton fiber elongation is regulated in a complex, orderly manner involving multiple genes and pathways. Prominent progress in molecular biology research on cotton fiber development is the isolation of some cotton fiber-specific or abundantly expressed genes. With the development of new technology and bioinformatics, expression profile analysis with RNA-seq technology as the main method has played an important role in cotton fiber development research. In this study, we used RNA-seq technology to sequence the transcriptome of seven different cotton tissues (root, leaf, anther, stigma, 7 DPA fiber, 14 DPA fiber, and 26 DPA fiber) to screen for highly abundant genes during fiber elongation and development.

In this study, the analysis of DEGs was performed from the transcriptome data of 7 DPA fiber and nonfiber tissues. There were 1,205 upregulated genes with significant expression differences found in 7 DPA fibers; many of these genes had been previously reported in fibers, such as *E6*^25^*, Flb2A*^26^*, GhAGP4*^27^*, GhFLA1*^28^*, GhEXPA*^29^*, GhMYB2*^30^*, GhACT1*^31^*, Rac1*^32^*, GhTUB1*^33^*,* and *GhCesA*^34^. GO enrichment analysis was performed on genes that were significantly upregulated in 7 DPA fibers. These gene products are mainly localized in the membrane, organelles, cell wall and other cell components and participate in molecular functions such as catalytic functions, binding, transport activity and molecular function regulation. The results were consistent with those reported by Qin^35^, Liu^36^ and Huang^37^, indicating that genes related to catalytic activity, lipid metabolism and the cell membrane may play an important role in the early elongation stage of fiber development. Subsequently, through analysis of KEGG metabolic pathways, it was found that the upregulated genes in fiber were mainly enriched in the categories of signal transduction, carbohydrate metabolism, protein translation and processing, transportation and catabolism, followed by energy metabolism, lipid metabolism, glycan biosynthesis and metabolism, cell growth and other metabolic pathways. This finding was consistent with the results reported by Fang et al., who found that carbohydrate metabolism, protein translation and processing, signal transduction and lipid metabolism played an important role in fiber elongation^38^. Similarly, DEGs were analyzed between fiber and nonfiber tissues at 14 DPA and 26 DPA, resulting in the identification of 1135 and 937 upregulated DEGs, respectively. The results of GO enrichment and KEGG functional analyses were similar to those of the cluster analysis of 7 DPA fibers, indicating that lipid metabolism, signal transduction, catalytic activity, the cell wall, and the cytoskeleton played important roles in the rapid and late elongation of cotton fibers.

### Fiber development-related transcription factors regulate cotton fiber elongation and development

A cotton fiber consists of a single epidermal cell of the ovule that undergoes specialized initial differentiation, elongation, thickening and dehydration maturation to form a mature epidermal fiber^39^. A large number of genes are required for fiber differentiation and development, but it is thus far unclear how these genes control and regulate fiber development. Transcription factors play an important regulatory role in the growth and evolution of cotton fiber. In this study, a total of 1,467 transcription factors were identified from families such as MYB, bHLH, bZIP, and TCP. Among these transcription factors, 148 were significantly upregulated in fibers. *GhMYB7*, an R2R3-MYB transcription factor, was highly expressed during fiber elongation. A cross-sectional assay of basal stems revealed that the cell wall thickness of vessels and interfascicular fibers was higher in transgenic lines overexpressing *GhMYB7* than in the wild type. This gene may be involved in regulating secondary cell wall biosynthesis in cotton fibers^40^. *GhMYB25* and *GhMYB25-like* were mainly expressed at high levels during the initial differentiation and early elongation of the fiber, and their expression decreased with the onset of rapid elongation of the fiber. The silencing of *GhMYB25* is associated with the production of short fibers in cotton^41^, while the suppression of *GhMYB25-like* produces fiber-less cotton^42^. *GhMYB46* showed the highest expression in 20 DPA fibers. Overexpression of *GhMYB46* leads to ectopic secondary cell wall (SCW) deposition in transgenic plants and could activate the promoter of the SCW cellulose synthase gene to regulate the elongation and development of cotton fiber^43^. *GhMYB109* is specifically expressed in the differentiation and elongation stages of cotton fiber primordial cells, and antisense-mediated suppression of *GhMYB109* leads to a substantial reduction of fiber length^44^. *GhDEL65*, a transcription factor of the bHLH protein family from upland cotton, is a homologous gene of *Arabidopsis GLABRA3* (*GL3*), which is expressed at a significant level in the early stage of fiber elongation; ectopic expression partially rescues hair body development. Ectopic expression of *GhDEL65* partially rescues the development of trichomes in the *Arabidopsis gl3* mutant^45^. *GhFSN1*, one of the transcription factors of the NAC family, has been found to exhibit high levels of transcript accumulation in 15–28 DPA fibers, but the expression of this gene is undetectable or very weak in other tissues of cotton. This gene regulates the formation of the SCW in cotton fiber^46^. *GhTCP14a*, a TCP transcription factor, is expressed at a high level during the rapid elongation period (6–12 DPA) of cotton fibers and regulates the rapid elongation development of the cotton fiber^47^. In this study, a large number of transcription factors were expressed either preferentially or specifically in cotton fibers. However, further research is required to determine how transcription factors regulate the elongation and development of cotton fibers.

### Fiber development-related functional genes regulate cotton fiber elongation and development

Researchers have successfully cloned and identified many cotton genes with fiber-dominant expression and studied their gene functions. These genes were all in the high-abundance expression database of fibers screened in this study (Table S3). For example, *GhTUB1* is preferentially expressed in the elongation and development stages of fibers^33^. *GhACT1* is mainly expressed in fiber cells, and its suppression disrupts the actin cytoskeleton and causes reduced fiber elongation, indicating that *GhACT1* plays an important role in the period of fiber elongation but does not play a role in the initial period of fiber cell development^31^. *GhPEL76* is a pectate lyase-like gene. Expression analysis (qRT-PCR) results showed that *GhPEL76* is mainly expressed in cotton fibers, and its expression levels are significantly different in long- and short-fiber varieties. Virus-induced *GhPEL76* silencing shortens fiber length, suggesting that *GhPEL76* has a positive regulatory effect on fiber elongation^48^. *GhLTPG1*, a GPI-anchored lipid transporter gene, was cloned from *Gossypium hirsutum* and shown to be significantly expressed in the period of rapid fiber elongation. After heterologous expression in *Arabidopsis*, the number of leaf epidermal hairs was significantly increased. In contrast, silencing of the *GhLTPG1* gene in upland cotton by using RNAi technology resulted in a significantly shortened fiber length, reduced polar lipid content and inhibited expression of genes related to fiber elongation^49^. *GhFIM2* is an actin gene that is expressed predominantly in the overlapping portion of the fiber elongation and SCW synthesis phases. In cotton, overexpression of the *GhFIM2* gene increases the expression level in the actin bundle in the fiber elongation period and accelerates the rate of fiber elongation so that the length of mature fibers is increased^50^. *GhEXPA8* is an *EXPANSIN* family gene that is expressed at a high level during the rapid elongation (7–25 DPA) of fiber. Overexpression of *GhEXPA8* can increase the fiber length and mark value to enhance fiber tenacity^51^. In this study, a large number of genes were screened for significantly high expression levels in the fiber elongation and development period, providing many new gene resources for genetic engineering for cotton fiber quality improvement.

### Analysis of highly abundant expressed genes in fiber elongation development

Through analysis of fibers and nonfibers transcriptome data, a total of 1324 highly expressed genes in fiber were screened, of which 330, 128, and 278 genes were predominantly expressed in 7, 14 and 26 dpa fibers. A total of 407 genes had clear functional annotations, but only a few genes have been reported in research. After sorting out the data and analyses, we found a large number of unreported new genes that are expressed in high abundance in fibers (Table S4). For example, *CotAD_24107* belongs to the proline-rich cell wall protein family genes, and its homologous gene *PdPRP* is preferred in immature poplar. Overexpression of *PdPRP* promotes secondary wall deposition and induces the expression of genes involved in microfibril angle and secondary wall biosynthesis^52^. In this study, we found that this gene was expressed in high abundance in cotton fibers, especially in the period of fiber secondary wall thickening (14 DPA). *CotAD_27598* is a protodermal factor gene. In Arabidopsis, *AtPDF2* is specifically expressed in bud epidermal cells and plays an indispensable role in bud differentiation^53,54^. This study showed that this gene was expressed in extremely high abundance during the differentiation stage of fibroblasts (7 DPA), and it was speculated that it played an important role in the initial stage of fibroblast development. The homologous gene *MtKCS* of *CotAD_37982* was predominantly expressed in the epidermal cells of the bud apical meristem, leaf primordium, and floral organs of *Medicago truncatula* and was mainly involved in the biosynthesis of very-long-chain fatty acids. However, overexpression of very-long-chain fatty acids can enhance the biosynthesis of cytokinins and promote the process of cell differentiation^55^. In this study, it was found that this gene was specifically expressed in fibers and was expressed in high abundance during the differentiation period of fiber primordial cells (7 DPA). It was speculated that this gene had the function of promoting fibroblast differentiation. *CotAD_37677* is a bidirectional sugar transporter gene. Its homologous gene *OsSWEET3a* functions as a glucose transporter and is predominantly expressed in the basic vascular bundles of rice seedlings^56^. This gene was mainly secondary to cotton fiber development. It was expressed in abundance during the wall thickening period (26 DPA), and we speculated that it transported glucose to fiber cells for cellulose biosynthesis. *CotAD_60133* encoded a skewing-related protein. Its homologous gene *AtSPR1* is related to directional cell expansion and functions by regulating cortical microtubule dynamics^57,58^, indicating that this gene may be regulated by microtubule dynamics to regulate the expansion and change of fiber cells. In conclusion, this study used transcriptome data analysis of fiber and nonfiber tissues to screen a large number of fiber dominant expression genes, which can be used as candidate genes for the improvement of cotton fiber quality.

To further verify the comprehensiveness and reliability of the transcriptome sequencing data from different cotton tissues obtained in this study, we selected 36 genes from the 7, 14 and 26 DPA genes that were significantly upregulated in the fiber to analyze the expression patterns among different cotton tissues by qRT-PCR. The significantly upregulated genes identified in fiber were all specifically or predominantly expressed in fiber. Among these genes, *CotAD_46044 (E6)*^25^, *CotAD_46959 (GhPRP1)*^59^, *CotAD_27919 (GhEXPA)*^51^, *CotAD_14327 (GhGASL3)*^60^, *CotAD_63563 (GhGLP1)*^61^, *CotAD_05318 (GhXTH7)*^62^, *CotAD_01886 (GhPEL)*^63^, *CotAD_49061 (GhKCS)*^64^, *CotAD_20528 (GhFb)*^26^, *CotAD_69249 (GhFLA7)* and *CotAD_1612 (GhFLA12)*^37^ have been previously reported to be specifically or preferentially expressed in the period of fiber elongation and secondary wall thickening and play an important role in cotton fiber elongation. The remaining 25 genes that were specifically or predominantly expressed in fiber had not been previously reported, so they could be used as candidate genes for further functional studies. By comparing the expression patterns of the selected fiber-upregulated genes between different tissues according to the qRT-PCR and RNA-seq data, we found that the gene expression patterns revealed by the two types of analysis were very similar. Therefore, the transcriptome libraries of different tissues constructed in this study were comprehensive and reliable and provided new genetic resources for the genetic engineering of cotton fiber quality improvement.

## Materials and methods

### Plant materials

Coker 312 was upland cotton cultivar and preserved in our laboratory, which was commonly used as test material in cotton research. In our study, using Coker 312 as material to identify the genes preferentially expressed in fiber development in *Gossypium hirsutum*. Our research contents complied with local and national regulations. Coker 312 was planted in a greenhouse. The roots and leaves were collected at 15, 25, and 35 days after germination, and the surface of the materials was cleaned with ddH_2_O. The root materials from the three periods were mixed together as the root tissue material and wrapped with aluminum foil, and the leaf material was subjected to the same procedure. The material was frozen in liquid nitrogen for 5 min and then transferred to a − 80 °C refrigerator for storage. Anthers were collected from cotton at − 3 ~ 0 DPA, stigmas were collected on the day of flowering, and fibers were collected at 7, 14, and 26 DPA (removing ovules). These samples were quickly placed in liquid nitrogen for freezing treatment for approximately 10 min and then transferred to a − 80 °C freezer for storage.

### RNA extraction, cDNA library construction and RNA sequencing

Total RNA was extracted from each sample using the RNAprep Pure Plant Kit (Polysaccharides & Polyphenolics-rich) (TIANGEN, Beijing, China) following the manufacturer’s protocol. We collected 14 samples from two biological replicates of each sample. Electrophoresis in a 1% agarose gel was used to determine whether the total RNA presented genomic DNA contamination, degradation or impurity. The concentration, purity and RNA integrity of the total RNA were further determined using a Kaiao K5500 spectrophotometer (Kaiao, Beijing, China) and an Agilent 2100 Bioanalyzer (Agilent Technologies, CA, USA). After the total RNA samples were qualified, oligo (dT) magnetic beads were used to enrich the mRNA. Fragmentation buffer (Agilent, CA, USA) was added to the obtained mRNA to generate short fragments. Then, the first strand of cDNA was synthesized with six-base random primers, and the second strand of cDNA was synthesized by adding buffer, dNTPs, RNase H and DNA polymerase I (NEB, MA, USA). The QIAQuick PCR kit was used for purification, and elution was conducted with EB buffer (QIAGEN, Germany). The purified double-stranded cDNA was then treated by terminal repair and A base and sequencing adapter addition (Illumina, CA, USA). Subsequently, a fragment of approximately 150 bp was recovered by agarose gel electrophoresis, and PCR amplification was performed to complete the library preparation. Finally, the constructed cDNA libraries were sequenced on the Illumina HiSeq 2000 platform.

### Bioinformatics analysis of RNA-seq data to identify DEGs

The initial results of transcriptome sequencing were in the form of original images, which could be employed for base recognition and transformation using CASAVA (v1.8) software to obtain the original sequence data^65^. The quality of the original sequence was evaluated by detecting the base sequencing error rate and G+C distribution. Higher-quality clean reads were obtained by collating and filtering to remove adaptor tags, reads with an N content greater than 5% and lower-quality ultrashort reads. In this study, the whole-genome data of *Gossypium hirsutum* TM-1 were used as a reference genome sequence (http://grand.cricaas.com.cn/home), and clean reads from seven different tissues were used for genome location analysis using TopHat2 software^66^. StringTie software was used to reassemble all clean reads for the prediction of new transcripts^67^. Gene expression level quantification was estimated via the FPKM (fragments per kilobase of transcript sequence per million fragments mapped) method using HTSeq software^68^. DEGseq software was used to identify the DEGs of different tissues according to a p value ≤ 0.05 and |Log2(Fold Change)| ≥ 2^69^. Finally, the statistical results for the DEGs among tissues were obtained.

### Functional classification of DEGs

The functional analysis of DEGs was conducted with GO and KEGG annotation. The GOseq R (v4.0.2) software package^70^ (https://www.r-project.org/)was used for the GO enrichment analysis of DEGs. We used KOBAS (v3.0) software (http://kobas.cbi.pku.edu.cn/kobas3/) to test the statistical abundance of DEGs in the KEGG database. The GO terms and KEGG pathways with corrected p values ≤ 0.05 were considered the thresholds to determine the significant enrichment of DEGs. The main functions and metabolic pathways of DEGs were preliminarily hypothesized.

### Verification of RNA-seq results by qRT-PCR

To verify the accuracy of the obtained differential gene expression patterns between fiber and nonfiber tissues, we screened 36 genes that were significantly upregulated in fibers for verification analysis by qRT-PCR. These DEG-specific primers were designed with the Primer3 online tool (http://bioinfo.ut.ee/primer3-0.4.0/) (Table S5). According to the manufacturer's instructions, cDNA synthesis was performed from 1 μg of total RNA in a 20 μL reaction mixture using a PrimerScript RT kit (TAKARA, Dalian, China). The 20 μL reactions were performed using 10 μL of SYBR Premix Ex Taq II (TLi RanseH Plus) (TAKARA, Dalian, China), 0.8 μL of 10 mM forward and reverse primers each, 7.4 µL of ddH_2_O and 1 μL of cDNA template, after which amplification reactions were conducted. The cotton Sad1 gene was used as an internal reference gene. The qRT-PCR conditions were as follows: 95 °C for 30 s, 40 cycles of 95 °C for 5 s and 60 °C for 34 s. Three biological and technical replicates were performed for each sample to verify the results of the qRT-PCR test, and relative gene expression levels were quantified via the 2-ΔΔCt method*.*

## Supplementary Information

Supplementary Figure S1.

Supplementary Figure S2.

Supplementary Figure S3.

Supplementary Figure S4.

Supplementary Figure S5.

Supplementary Table S1.

Supplementary Table S2.

Supplementary Table S3.

Supplementary Table S4.

Supplementary Table S5.

## References

[1] Yoo MJ, Wendel JF (2014). Comparative evolutionary and developmental dynamics of the cotton (*Gossypium Hirsutum*) fiber transcriptome. PLoS Genet..

[2] Kim HJ, Triplett BA (2001). Cotton fiber growth in planta and in vitro: Models for plant cell elongation and cell wall biogenesis. Plant Physiol..

[3] McCombie WR, McPherson JD, Mardis ER (2019). Next-generation sequencing technologies. Cold Spring Harb. Perspect. Med..

[4] Arya SK, Dhar YV, Upadhyay SK, Asif MH, Verma PC (2018). De novo characterization of phenacoccus solenopsis transcriptome and analysis of gene expression profiling during development and hormone biosynthesis. Sci. Rep..

[5] Filichkin SA (2010). Genome-wide mapping of alternative splicing in *Arabidopsis Thaliana*. Genome Res..

[6] Quesada T (2008). Comparative analysis of the transcriptomes of *Populus Trichocarpa* and *Arabidopsis Thaliana* suggests extensive evolution of gene expression regulation in *Angiosperms*. New Phytol..

[7] Severin AJ (2010). RNA-seq atlas of glycine max: A guide to the *Soybean* transcriptome. BMC Plant Biol..

[8] Fu C (2017). Transcriptomic analysis reveals new insights into high-temperature-dependent glume-unclosing in an elite rice male sterile line. Front. Plant Sci..

[9] Shu Y, Li W, Zhao J, Liu Y, Guo C (2018). Transcriptome sequencing and expression profiling of genes involved in the response to abiotic stress in *Medicago Ruthenica*. Genet. Mol. Biol..

[10] Zhang B (2018). A combined small RNA and transcriptome sequencing analysis reveal regulatory roles of miRNAs during anther development of upland cotton carrying cytoplasmic male sterile *Gossypium Harknessii* (D2) cytoplasm. BMC Plant Biol..

[11] Jian H (2019). Joint QTL mapping and transcriptome sequencing analysis reveal candidate flowering time genes in *Brassica Napus *L. BMC Genomics.

[12] Odintsova TI (2019). Defensin-like peptides in wheat analyzed by whole-transcriptome sequencing: A focus on structural diversity and role in induced resistance. PeerJ.

[13] Qian Y, Ren Q, Zhang J, Chen L (2019). Transcriptomic analysis of the maize (*Zea Mays* L.) inbred line B73 response to heat stress at the seedling stage. Gene.

[14] Cao A (2017). Comparative transcriptome analysis of SE initial dedifferentiation in cotton of different SE capability. Sci. Rep..

[15] Parekh MJ, Kumar S, Fougat RS, Zala HN, Pandit RJ (2018). Transcriptomic profiling of developing fiber in levant cotton (*Gossypium Herbaceum* L.). Funct. Integr. Genomics..

[16] Hamid R, Marashi H, Tomar RS, Malekzadeh SS, Sabara PH (2019). Transcriptome analysis identified aberrant gene expression in pollen developmental pathways leading to CGMS in cotton (*Gossypium Hirsutum* L.). PLoS ONE.

[17] Padmalatha KV (2012). Genome-wide transcriptomic analysis of cotton under drought stress reveal significant down-regulation of genes and pathways involved in fibre elongation and up-regulation of defense responsive genes. Plant Mol. Biol..

[18] Li PT (2017). Comparative transcriptome analysis of cotton fiber development of upland cotton (*Gossypium Hirsutum*) and chromosome segment substitution lines from *G. Hirsutum* x *G. Barbadense*. BMC Genomics.

[19] Hu H (2018). Transcriptomic repertoires depict the initiation of lint and fuzz fibres in cotton (*Gossypium Hirsutum* L.). Plant Biotechnol. J..

[20] Xu Y (2019). Deep transcriptome analysis reveals reactive oxygen species (ROS) network evolution, response to abiotic stress, and regulation of fiber development in cotton. Int. J. Mol. Sci..

[21] Wan Q, Zhang H, Ye W, Wu H, Zhang T (2014). Genome-wide transcriptome profiling revealed cotton fuzz fiber development having a similar molecular model as *Arabidopsis Trichome*. PLoS ONE.

[22] Man W (2016). A Comparative transcriptome analysis of two sets of backcross inbred lines differing in lint-yield derived from a *Gossypium Hirsutum* x *Gossypium Barbadense* Population. Mol. Genet. Genomics..

[23] Li X (2017). A genome-wide analysis of the small auxin-up RNA (SAUR) gene family in cotton. BMC Genomics.

[24] Yang L (2005). Validation of a cotton-specific gene, *Sad1*, used as an endogenous reference gene in qualitative and real-time quantitative PCR detection of transgenic cottons. Plant Cell Rep..

[25] John ME, Crow LJ (1992). Gene expression in cotton (*Gossypium Hirsutum* L.) fiber: Cloning of the mRNAs. Proc. Natl. Acad. Sci. USA..

[26] Rinehart JA, Petersen MW, John ME (1996). Tissue-specific and developmental regulation of cotton gene FbL2A: Demonstration of promoter activity in transgenic plants. Plant Physiol..

[27] Li Y (2010). Suppression of *GhAGP4* gene expression repressed the initiation and elongation of cotton fiber. Plant Cell Rep..

[28] Huang GQ (2013). A fasciclin-like arabinogalactan protein, GhFLA1, is involved in fiber initiation and elongation of cotton. Plant Physiol..

[29] Harmer SE, Orford SJ, Timmis JN (2002). Characterisation of six alpha-expansin genes in *Gossypium Hirsutum* (upland cotton). Mol. Genet. Genomics..

[30] Wang S (2004). Control of plant trichome development by a cotton fiber *MYB* gene. Plant Cell.

[31] Li XB, Fan XP, Wang XL, Cai L, Yang WC (2005). The cotton *ACTIN1* gene is functionally expressed in fibers and participates in fiber elongation. Plant Cell.

[32] Kim HJ, Triplett BA (2004). Characterization of GhRac1 GTPase expressed in developing cotton (*Gossypium Hirsutum* L.) fibers. Biochim. Biophys. Acta..

[33] Li XB, Cai L, Cheng NH, Liu JW (2002). Molecular characterization of the cotton *GhTUB1* gene that is preferentially expressed in fiber. Plant Physiol..

[34] Li A (2013). An integrative analysis of four CESA isoforms specific for fiber cellulose production between *Gossypium Hirsutum* and *Gossypium Barbadense*. Planta.

[35] Qin YM, Zhu YX (2011). How cotton fibers elongate: A tale of linear cell-growth mode. Curr. Opin. Plant Biol..

[36] Liu K, Sun J, Yao L, Yuan Y (2012). Transcriptome analysis reveals critical genes and key pathways for early cotton fiber elongation in ligon lintless-1 mutant. Genomics.

[37] Huang GQ (2008). Characterization of 19 novel cotton *FLA* genes and their expression profiling in fiber development and in response to phytohormones and salt stress. Physiol Plant..

[38] Fang L (2014). Cotton fiber elongation network revealed by expression profiling of longer fiber lines introgressed with different *Gossypium Barbadense* chromosome segments. BMC Genomics.

[39] Haigler CH, Betancur L, Stiff MR, Tuttle JR (2012). Cotton fiber: A powerful single-cell model for cell wall and cellulose research. Front. Plant Sci..

[40] Huang J, Chen F, Wu S, Li J, Xu W (2016). Cotton *GhMYB7* is predominantly expressed in developing fibers and regulates secondary cell wall biosynthesis in transgenic *Arabidopsis*. Sci. China Life Sci..

[41] Machado A, Wu Y, Yang Y, Llewellyn DJ, Dennis ES (2009). The MYB transcription factor *GhMYB25* regulates early fibre and trichome development. Plant J..

[42] Walford SA, Wu Y, Llewellyn DJ, Dennis ES (2011). *GhMYB25-like*: A key factor in early cotton fibre development. Plant J..

[43] Huang J (2019). Genome-wide identification of R2R3-MYB transcription factors regulating secondary cell wall thickening in cotton fiber development. Plant Cell Physiol..

[44] Pu L, Li Q, Fan X, Yang W, Xue Y (2008). The R2R3 MYB transcription factor *GhMYB109* is required for cotton fiber development. Genetics.

[45] Shangguan XX, Yang CQ, Zhang XF, Wang LJ (2016). Functional characterization of a basic helix-loop-helix (bHLH) transcription factor *GhDEL65* from cotton (*Gossypium Hirsutum*). Physiol. Plant..

[46] Zhang J (2018). The cotton (*Gossypium Hirsutum)* NAC transcription factor (*FSN1*) as a positive regulator participates in controlling secondary cell wall biosynthesis and modification of fibers. New Phytol..

[47] Li W (2017). Genome-wide identification and characterization of *TCP* transcription factor genes in upland cotton (*Gossypium Hirsutum*). Sci. Rep..

[48] Sun H (2020). Pectate lyase-like gene *GhPEL76* regulates organ elongation in *Arabidopsis* and fiber elongation in cotton. Plant Sci..

[49] Deng T (2016). GhLTPG1, a cotton GPI-anchored lipid transfer protein, regulates the transport of phosphatidylinositol monophosphates and cotton fiber elongation. Sci. Rep..

[50] Zhang M (2017). Overexpression of GhFIM2 propels cotton fiber development by enhancing actin bundle formation. J. Integr. Plant Biol..

[51] Bajwa KS (2015). Stable transformation and expression of *GhEXPA8* fiber expansin gene to improve fiber length and micronaire value in cotton. Front. Plant Sci..

[52] Li S (2019). Proline-rich protein gene *PdPRP* regulates secondary wall formation in poplar. J. Plant Physiol..

[53] Demko V, Ako E, Perroud PF, Quatrano R, Olsen OA (2016). The phenotype of the CRINKLY4 deletion mutant of physcomitrella patens suggests a broad role in developmental regulation in early land plants. Planta.

[54] Kamata N, Okada H, Komeda Y, Takahashi T (2013). Mutations in epidermis-specific HD-ZIP IV genes affect floral organ identity in *Arabidopsis Thaliana*. Plant J..

[55] Yang T (2021). The 3-ketoacyl-CoA synthase WFL is involved in lateral organ development and cuticular wax synthesis in *Medicago Truncatula*. Plant Mol. Biol..

[56] Morii M (2020). The dual function of *OsSWEET3a* as a gibberellin and glucose transporter is important for young shoot development in rice. Plant Cell Physiol..

[57] Califar B, Sng NJ, Zupanska A, Paul AL, Ferl RJ (2020). Root skewing-associated genes impact the spaceflight response of *Arabidopsis Thaliana*. Front. Plant Sci..

[58] Nakajima K, Furutani I, Tachimoto H, Matsubara H, Hashimoto T (2004). *SPIRAL1* encodes a plant-specific microtubule-localized protein required for directional control of rapidly expanding *Arabidopsis* cells. Plant Cell.

[59] Tan H, Creech RG, Jenkins JN, Chang YF, Ma DP (2001). Cloning and expression analysis of two cotton (*Gossypium Hirsutum* L.) genes encodingcell wall proline-rich proteins. DNA Seq..

[60] Liu ZH (2013). Cotton GASL genes encoding putative gibberellin-regulated proteins are involved in response to GA signaling in fiber development. Mol. Biol. Rep..

[61] Kim HJ, Triplett BA (2004). Cotton fiber germin-like protein. I. Molecular cloning and gene expression. Planta.

[62] Lee J (2010). Xyloglucan endotransglycosylase/hydrolase genes in cotton and their role in fiber elongation. Planta.

[63] Sun H (2018). Genome-wide identification and expression analyses of the pectate lyase (PEL) gene family in cotton (*Gossypium Hirsutum* L.). BMC Genomics.

[64] Xiao GH, Wang K, Huang G, Zhu YX (2016). Genome-scale analysis of the cotton *KCS* gene family revealed a binary mode of action for gibberellin a regulated fiber growth. J. Integr. Plant Biol..

[65] Whiteford N (2009). Swift: Primary data analysis for the illumina solexa sequencing platform. Bioinformatics.

[66] Kim D (2013). TopHat2: Accurate alignment of transcriptomes in the presence of insertions deletions and gene fusions. Genome Biol..

[67] Pertea M (2015). StringTie enables improved reconstruction of a transcriptome from RNA-seq reads. Nat. Biotechnol..

[68] Trapnell C (2010). Transcript assembly and quantification by RNA-seq reveals unannotated transcripts and isoform switching during cell differentiation. Nat. Biotechnol..

[69] Wang L, Feng Z, Wang X, Wang X, Zhang X (2010). DEGseq: An R package for identifying differentially expressed genes from RNA-seq Data. Bioinformatics.

[70] Wickham H (2011). The split-apply-combine strategy for data analysis. J. Stat. Softw..

