# Supplementary material for: Comparative transcriptome analysis of fiber and nonfiber tissues to identify the genes preferentially expressed in fiber development in Gossypium hirsutum
Source: Sci Rep. 2021 Nov 24;11:22833. doi: 10.1038/s41598-021-01829-8 (PMC8613186; doi:10.1038/s41598-021-01829-8)
Supplement: Supplementary file 1 — Supplementary Figure S1. [file 41598_2021_1829_MOESM1_ESM.pdf]

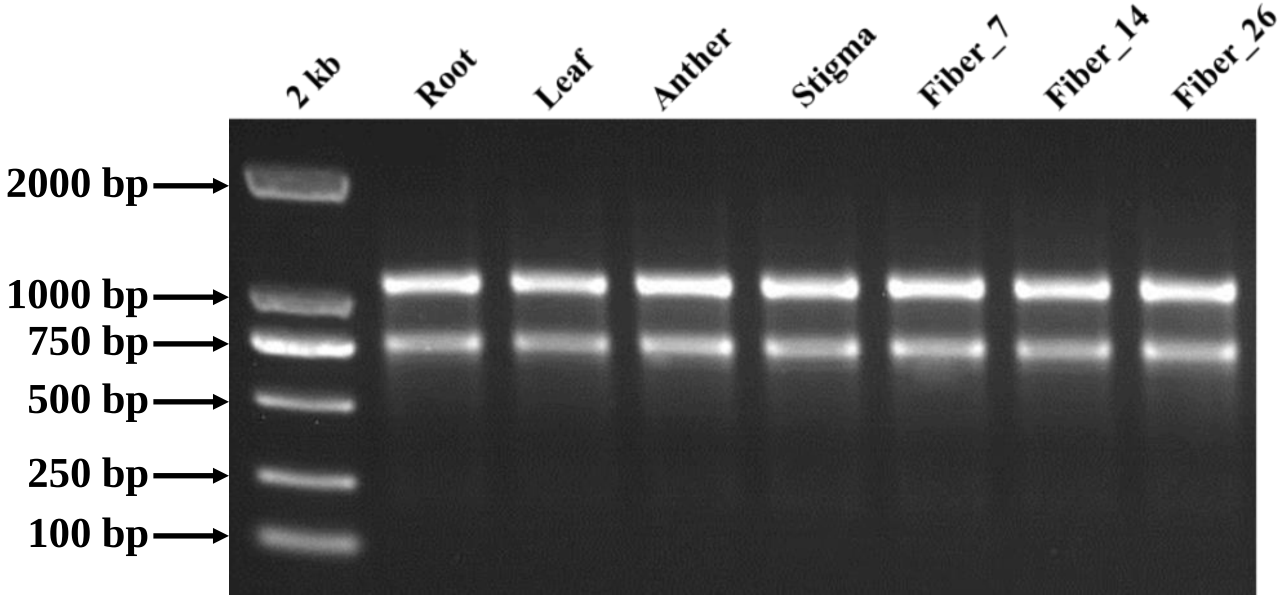

Figure S1. Total RNA electropherogram for seven different tissues of cotton

2 kb: 2 kb maker; Root: root; Leaf: leaf; Anther: anther; Stigma: stigma;

Fiber_7: 7 DPA fiber; Fiber_14: 14 DPA fiber; Fiber_26: 26 DPA fiber
