# Supplementary figures and images for: Comparative transcriptome analysis of fiber and nonfiber tissues to identify the genes preferentially expressed in fiber development in Gossypium hirsutum
Source: Sci Rep. 2021 Nov 24;11:22833. doi: 10.1038/s41598-021-01829-8 (PMC8613186; doi:10.1038/s41598-021-01829-8)

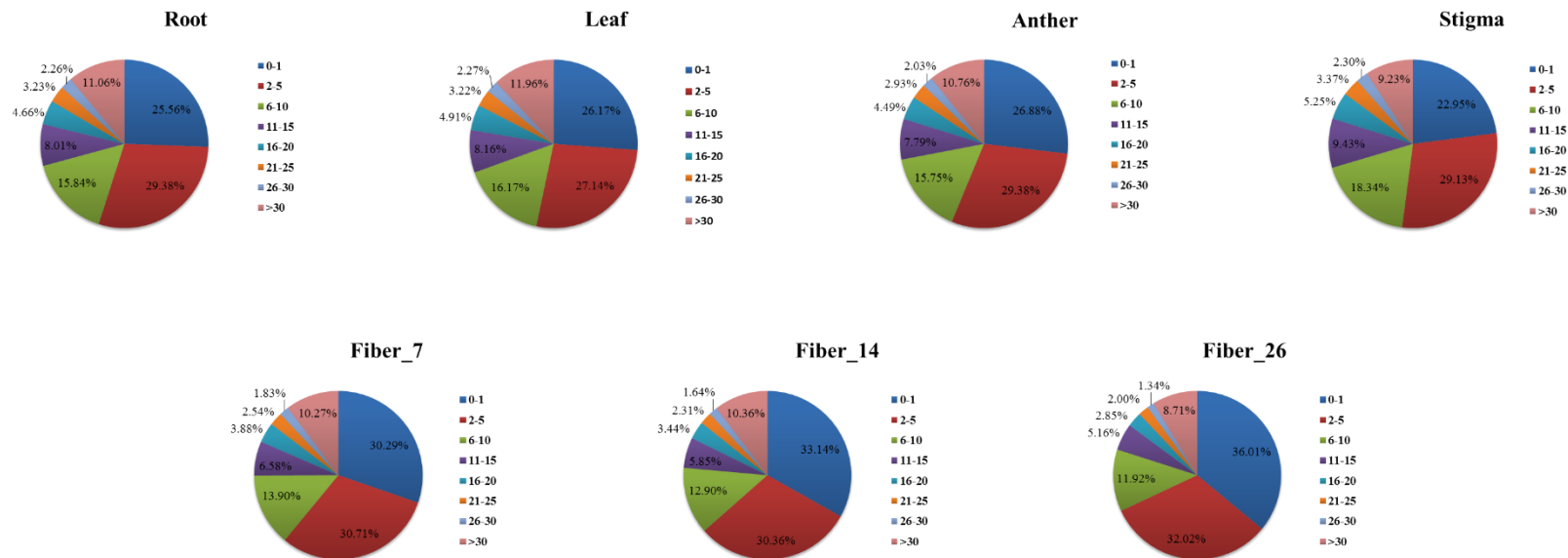

Figure S2. Transcript coverage depth statistics for each tissue

Supplement: Supplementary file 2 — Supplementary Figure S2. [file 41598_2021_1829_MOESM2_ESM.pdf]
