# Supplementary material for: Comparative transcriptome analysis of fiber and nonfiber tissues to identify the genes preferentially expressed in fiber development in Gossypium hirsutum
Source: Sci Rep. 2021 Nov 24;11:22833. doi: 10.1038/s41598-021-01829-8 (PMC8613186; doi:10.1038/s41598-021-01829-8)
Supplement: Supplementary file 4 — Supplementary Figure S4. [file 41598_2021_1829_MOESM4_ESM.pdf]

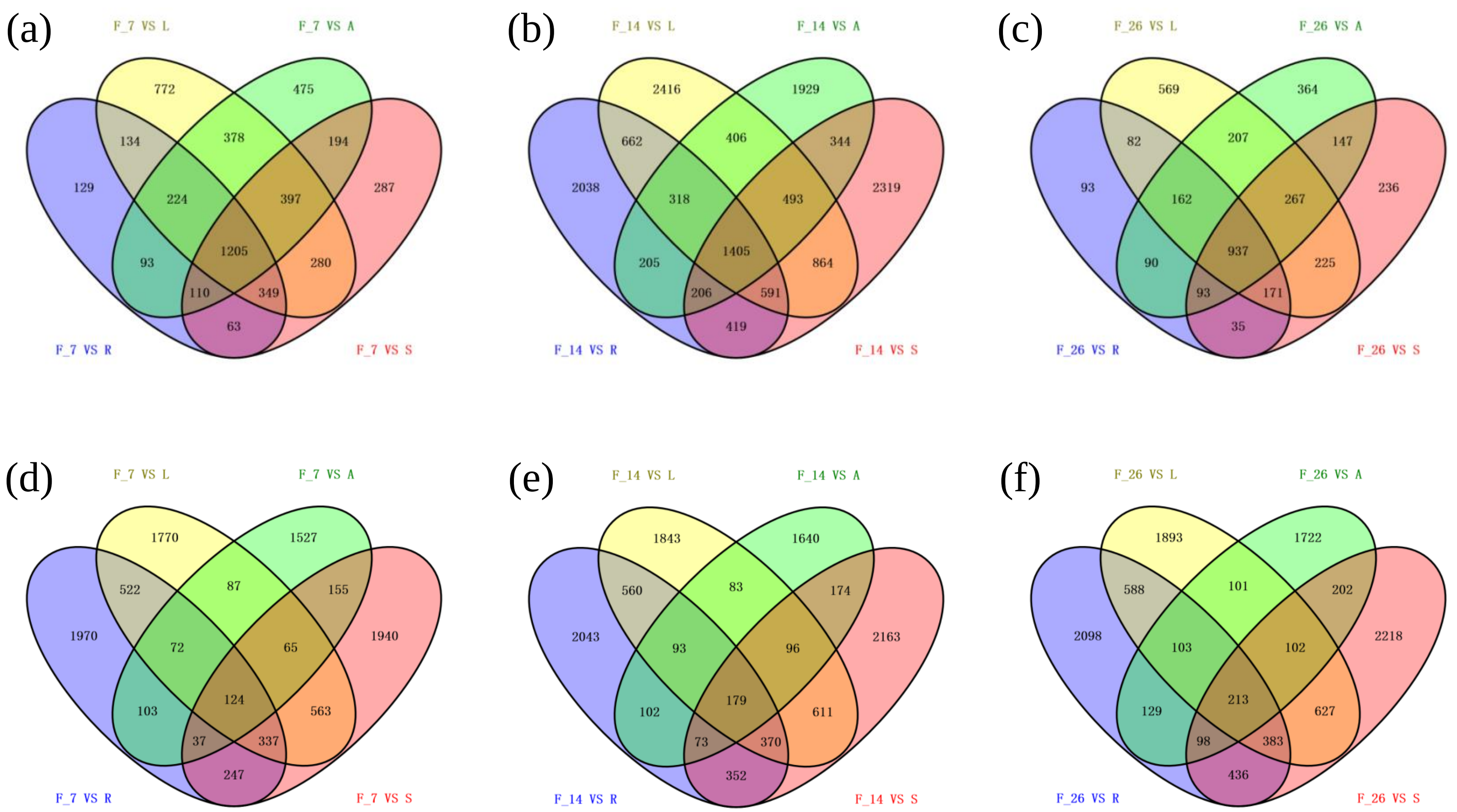
(a)
F_7 VS L
F_7 VS A
F_7 VS R
F_7 VS S
772
475
134
378
194
129
224
397
287
1205
93
280
110
349
63
(b)
F_14 VS L
F_14 VS A
F_14 VS R
F_14 VS S
2416
1929
662
406
344
2038
318
493
2319
1405
205
864
206
591
419
(c)
F_26 VS L
F_26 VS A
F_26 VS R
F_26 VS S
569
364
82
207
147
93
162
267
236
937
90
225
93
171
35
(d)
F_7 VS L
F_7 VS A
F_7 VS R
F_7 VS S
1770
1527
522
87
155
1970
72
65
1940
124
103
563
37
337
247
(e)
F_14 VS L
F_14 VS A
F_14 VS R
F_14 VS S
1843
1640
560
83
174
2043
93
96
2163
179
102
611
73
370
352
(f)
F_26 VS L
F_26 VS A
F_26 VS R
F_26 VS S
1893
1722
588
101
202
2098
103
102
2218
213
129
627
98
383
436

Figure S4. Comparative analysis of up- and down-regulated DEGs between fiber and nonfiber tissues.

(a, b, c): Venn diagram analysis of upregulated DEGs in 7 DPA, 14 DPA and 26 DPA fibers; (d, e, f): Venn diagram analysis of downregulated DEGs in 7 DPA, 14 DPA and 26 DPA fibers.
