# Supplementary material for: Comparative transcriptome analysis of fiber and nonfiber tissues to identify the genes preferentially expressed in fiber development in Gossypium hirsutum
Source: Sci Rep. 2021 Nov 24;11:22833. doi: 10.1038/s41598-021-01829-8 (PMC8613186; doi:10.1038/s41598-021-01829-8)
Supplement: Supplementary file 5 — Supplementary Figure S5. [file 41598_2021_1829_MOESM5_ESM.pdf]

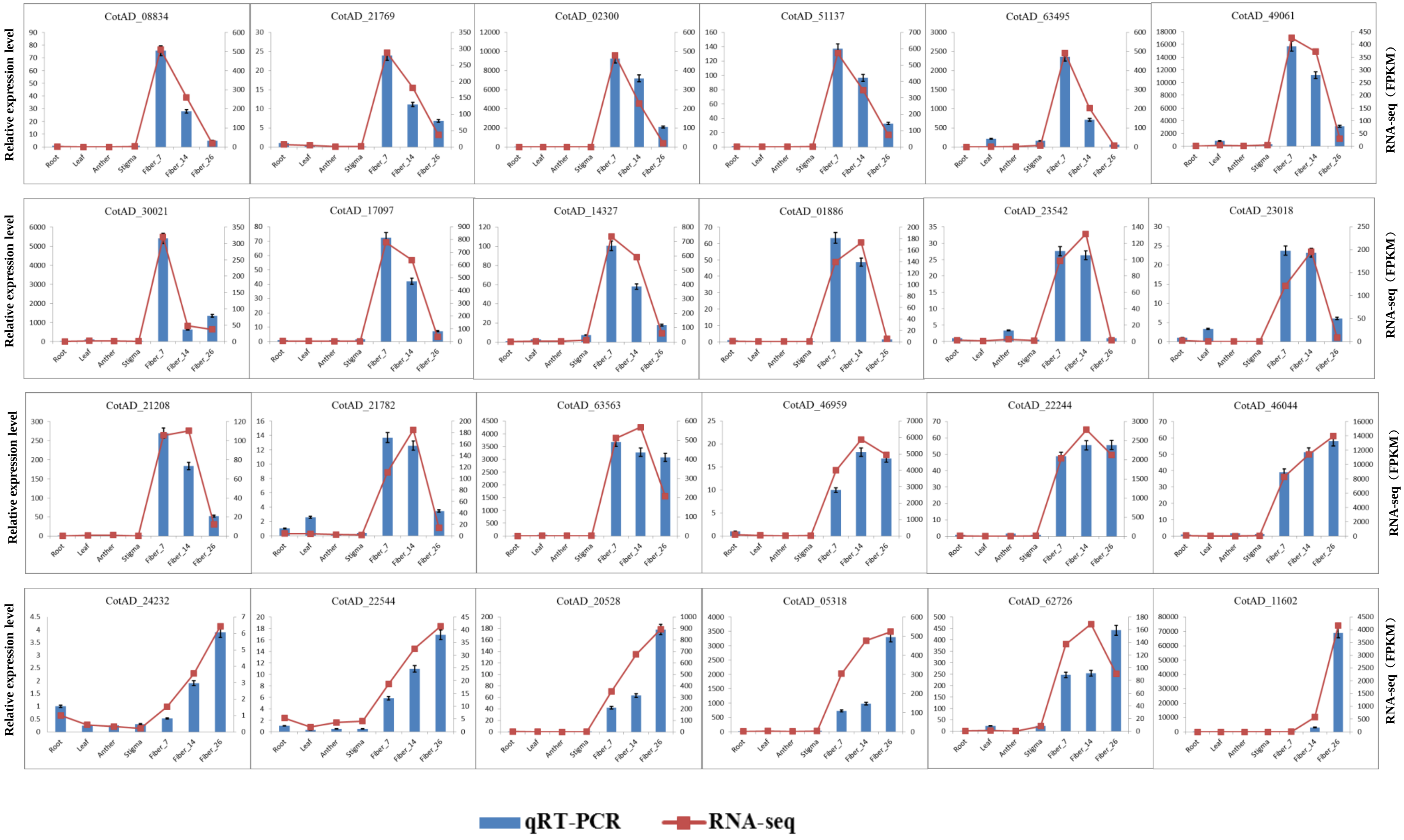

Figure S5. Validation of RNA-seq data by qRT-PCR.

Root: root; Leaf: leaf; Anther: anther; Stigma: stigma; Fiber_7: 7 DPA fiber; Fiber_14: 14 DPA fiber; Fiber_26: 26 DPA fiber; Columns indicate the results of qRT-PCR, and zigzag lines indicate the results of transcriptome sequencing.
