# Supplementary material for: Comparative transcriptome analysis of fiber and nonfiber tissues to identify the genes preferentially expressed in fiber development in Gossypium hirsutum
Source: Sci Rep. 2021 Nov 24;11:22833. doi: 10.1038/s41598-021-01829-8 (PMC8613186; doi:10.1038/s41598-021-01829-8)
Supplement: Supplementary file 6 — Supplementary Table S1. [file 41598_2021_1829_MOESM6_ESM.pdf]

Table S1. Total RNA quality in different tissues of cotton

**Sample name**	**Concentration (ng/μL)**	**Volume (μL)**	**Quantity (μg)**	**$OD_{260/230}$**	**$OD_{260/280}$**	**28S/18S***	**Integrity index***	**Standard***
Root	320	100	32.0	2.4	1.9	2.3	9.3	A
Leaf	250	100	25.0	2.2	1.9	2.1	8.4	A
Anther	214	100	21.4	2.3	1.9	2.2	8.6	A
Stigma	235	100	23.5	2.1	1.9	1.9	7.8	A
Fiber_7	324	100	32.4	2.2	1.9	2.0	8.1	A
Fiber_14	332	100	33.2	2.2	1.9	2.0	8.8	A
Fiber_26	204	100	20.4	2.3	1.9	2.2	7.1	A

Note: 28S/18S* is the index to measure the integrity of the extracted RNA. If 28S/18S is 1.9~2.3, it indicates that the integrity of the extracted RNA is good, and there is basically no degradation.

Integrity index*: RNA intergrity number

Standard*: Sample determination description

A. The samples are qualified and meet the requirements of database building;

B. Some indexes meet the requirements of database building, and it can be tried to build the database;

C. If the sample is unqualified, it is recommended to send the sample again.
