# Supplementary material for: Comparative transcriptome analysis of fiber and nonfiber tissues to identify the genes preferentially expressed in fiber development in Gossypium hirsutum
Source: Sci Rep. 2021 Nov 24;11:22833. doi: 10.1038/s41598-021-01829-8 (PMC8613186; doi:10.1038/s41598-021-01829-8)
Supplement: Supplementary file 7 — Supplementary Table S2. [file 41598_2021_1829_MOESM7_ESM.pdf]

Table S2. Distribution of transcript expression (FPKM) in each tissue

Sample	0-0.1 FI*	0.1-0.3 FI	0.3-3.57 FI	3.57-15 FI	15-60 FI	>60 FI	All genes
Root	5959(10.04%)	6691(11.27%)	28244(47.59%)	14036(23.65%)	3687(6.21%)	735(1.24%)	59352
Leaf	6603(11.42%)	6636(11.47%)	27617(47.74%)	12594(21.77%)	3573(6.18%)	820(1.42%)	57843
Anther	7217(11.50%)	7897(12.59%)	30524(48.64%)	12985(20.69%)	3372(5.37%)	754(1.20%)	62749
Stigma	6756(11.14%)	7204(11.87%)	29646(48.87%)	13455(22.18%)	3013(4.97%)	592(0.98%)	60666
Fiber_7	6865(12.43%)	7017(12.70%)	25862(46.82%)	11017(19.94%)	3529(6.39%)	952(1.72%)	55242
Fiber_14	7430(13.72%)	7740(14.29%)	25288(46.69%)	9439(17.43%)	3399(6.28%)	862(1.59%)	54158
Fiber_26	8126(15.46%)	8534(16.24%)	24749(47.08%)	7682(14.61%)	2704(5.14%)	770(1.46%)	52565

Note: FI*: FPKM Interval. Since there are certain differences in the number of gene expression and the distribution of gene expression values in the samples, the sample expression value (FPKM) can be divided into different intervals, and the number of genes expressed by the samples in different expression intervals can be calculated.
