# Supplementary material for: Comparative transcriptome analysis of fiber and nonfiber tissues to identify the genes preferentially expressed in fiber development in Gossypium hirsutum
Source: Sci Rep. 2021 Nov 24;11:22833. doi: 10.1038/s41598-021-01829-8 (PMC8613186; doi:10.1038/s41598-021-01829-8)
Supplement: Supplementary file 8 — Supplementary Table S3. [file 41598_2021_1829_MOESM8_ESM.pdf]

Table S3. The FPKM value Change of genes related to fiber elongation development in different tissues

Function category	Gene_name	Gene_id	FPKM value							Description
			Fiber_7	Fiber_14	Fiber_26	Root	Leaf	Anther	Stigma	
Cytoskeletal Components	*GhTUBA4*	CotAD_36056	496.39	1001.53	1014.82	33.17	1.21	57.25	52.82	tubulin alpha-4 chain
	GhTUBB6	CotAD_51888	71.86	404.22	446.42	2.26	1.66	0.03	0.03	Beta-6 tubulin
	GhTUB1	CotAD_75071	125.77	86.43	39.08	12.53	2.77	6.03	8.28	tubulin
	GhACT1	CotAD_57082	267.32	239.04	132.88	26.12	19.87	16.04	35.01	actin
	GhFIM1	CotAD_05357	146.21	187.84	194.19	47	17.07	19	38.6	actin
	GhTUBB2	CotAD_18492	153.92	169.44	151.54	15.49	6.21	9.64	12.13	tubulin beta-2 chain-like
	GhPSS1	CotAD_24245	74.62	82.5	63.39	2.01	0.68	4.68	5.01	kinesin-1-like protein PSS1
	GhCLC2	CotAD_46336	66.76	74.38	214.06	9.94	7.28	5.99	6.28	clathrin light chain 2-like
	GhHMGB3	CotAD_70969	34.17	74.24	60.68	3.78	2.5	5.86	6.19	high mobility group B protein 3-like
	GhEXPA8	CotAD_27919	287.42	468.93	356.36	49.14	35.78	22.76	23.63	expansin family gene
	GhDRP1	CotAD_19824	33.98	42.37	44.29	14.57	4.61	9.45	8.83	dynamin-related protein 1E
	GhDYN2	CotAD_15211	17.53	13.86	13.84	5.37	1.49	8.51	5.29	dynamin-2B
Glucose metabolism	*GhXTH23*	CotAD_05386	698.04	120.1	17.6	18.05	12.06	6.38	10.69	xyloglucan endotransglucosylase/hydrolase protein 23
	GhABFF	CotAD_31849	343.77	228.78	8.7	3.22	0.47	1.31	3.63	acid beta-fructofuranosidase-like
	GhADPG1	CotAD_28452	285.64	515.89	494.53	9.19	0.18	6.51	7.96	polygalacturonase ADPG1-like
	GhUGP1	CotAD_51418	208.38	201.27	181.74	6.56	6.27	4.48	3.01	UTP--glucose-1-phosphate uridylyltransferase
	GhGBA	CotAD_59939	167.66	462.31	733.3	0.06	0.02	0	0.11	lysosomal beta glucosidase
	GhPEL76	CotAD_56546	158.80	223.17	442.30	1.51	4.71	6.06	2.01	pectate lyase-like
	GhEG24	CotAD_53384	139.02	23.28	0.21	9.66	0.08	0.08	0.94	endoglucanase 24-like
	GhSUS	CotAD_06514	85.49	30.66	94.65	3.64	0.51	2.48	2.75	sucrose synthase-like
Fatty acid metabolism	*GhECR2*	CotAD_36666	439.94	290.29	80.23	7.91	3.83	2.24	6.42	very-long-chain enoyl-CoA reductase
	GhFAR3	CotAD_43773	125.76	23.45	0.55	0.72	0.72	0.46	0.55	fatty acyl-CoA reductase 3-like
	GhLACS1	CotAD_33757	81.88	18.12	4.67	1.69	6.18	0.38	2.16	long chain acyl-CoA synthetase 1
	GhLTPG1	CotAD_48107	67.85	146.61	89.23	5.68	3.48	6.13	1.96	GPI-anchored lipid transporter
	GhGDPDL3	CotAD_29790	78.94	72.72	12.15	10.55	4.13	3.93	5.38	glycerophosphoryl diester phosphodiesterase family protein
	GhSACPD9	CotAD_55670	78.91	61.81	26.5	5.82	2.27	1.66	3.66	stearoyl-[acyl-carrier-protein] 9-desaturase
	GhACC	CotAD_62937	69.04	21.85	7.04	7.96	1.28	3.36	4.02	acetyl-CoA carboxylase 1-like
	GhFAD2	CotAD_33272	143.87	213.76	26.25	25.61	29.46	31.53	13.18	delta (12)-fatty-acid desaturase
	GhHACD3	CotAD_49788	54.68	26.65	10.79	9.00	4.96	4.03	2.93	very-long-chain (3R)-3-hydroxyacyl-CoA dehydratase 2

Secondary metabolism	*GhHCT*	CotAD_55936	134.22	48.79	2.73	1.8	0.36	0.15	6.65	hydroxycinnamoyltransferase
	GhICDH	CotAD_51417	131.31	47.13	45.03	3.69	2.81	2.14	3.25	isocitrate dehydrogenase [NADP]
	GhMAH1	CotAD_27264	93.06	368.95	29.55	0	0.07	0.09	0.48	alkane hydroxylase MAH1
	GhF3'5'H	CotAD_16446	85.97	14.26	2.1	2.99	0.1	4.27	0.65	flavonoid 3',5'-hydroxylase 2
	GhACO1	CotAD_01574	68.76	94.17	83.07	3.16	1.86	3.08	1.89	1-aminocyclopropane-1-carboxylate oxidase 1-like
