# Supplementary material for: Comparative transcriptome analysis of fiber and nonfiber tissues to identify the genes preferentially expressed in fiber development in Gossypium hirsutum
Source: Sci Rep. 2021 Nov 24;11:22833. doi: 10.1038/s41598-021-01829-8 (PMC8613186; doi:10.1038/s41598-021-01829-8)
Supplement: Supplementary file 10 — Supplementary Table S5. [file 41598_2021_1829_MOESM10_ESM.pdf]

Table S5. The sequences of primers used in this study

Gene id	Forward primer (5′~3′)	Reverse primer (5′~3′)
Sad1	CCAAAGGAGGTGCCTGTTCA	TTGAGGTGAGTCAGAATGTTGTTC
CotAD_46044	CGTTCCCCAGACCACCCAAA	GGAGGATTCGGGGATGCTGT
CotAD_46959	TGGAAAGCCAAGACTCAATGATG	GTGGTGGTTTCACCTTGGGA
CotAD_27919	CTTCTACGGTGGTGCTGATGCTAC	AAGTTGGTGGCTGTCACGGTTATG
CotAD_22244	CCAAACCGGTTCGACAACTC	AATACTGTACGACAGGTGATCAGG
CotAD_98043	ACTGGCGTCAGCAGCAACATG	GTGCCACCGTTACCGTCTGATC
CotAD_14327	GGCTCAGCCTCGTTCACCTT	GGACACTCGTGTTTGTTCCCG
CotAD_51137	TCCCCTACTTATCTCACCACCA	GTTTTCATGGTCTTCTTCGCCG
CotAD_63563	GCAAGAAGACAGCCACCGAA	CTGAGGACATGAAACCGGCA
CotAD_08834	ACAGTGCTCAGTCCTCCGTACC	AGCAGCCAGAAGCAGTGAAGTG
CotAD_63495	CTGCTGCTGCTGGAATCAGAGAAG	AATGCCTGAAGCTGCTCAGTGTAC
CotAD_02300	GTTTGTCGCCGATGACCCTT	ATCCGCCATGTCTTCACTGC
CotAD_49061	CCTCCTGTCACTTGTCGTGTTCC	TCGCCTCTAGCAGCCTCCATG
CotAD_37838	CACGAGGAGTGGACATGCTAAGC	TGAGCGGCAATCTGTGAGTGTTAC
CotAD_20528	GCTGTGGCCATGTTTGTTGT	ATGCACCAAGAGAGCAATTAAAGAG
CotAD_30021	GCCTGCCACTAAATGTTGCAT	ACCTTGTGCTTGTGATTCGGT
CotAD_05318	AGATGCTGTGCGAGATGAGC	GCCTCCCACAGCGTTGAATA
CotAD_55936	ATTCATGCCATCTTCACCGTCACG	CGCCACGCAGTTCACACCAG
CotAD_21769	GAAGTCGCCGAATTAGCAACCAAC	CACTGAACCAAGTACCTCCGTCTG
CotAD_31849	CGATGGTTCCTGGCGGATTACTG	CCAAGTGCCGTCGTGTCAAGTC
CotAD_23413	GATCAGGCAGTAGTGTACGCAGTG	CGAAGCCGCAGAGCAAGGATC
CotAD_63265	AGAACTTGACAGCCGCCACTATTC	GGAACGTGCTCCGTTGATGACC
CotAD_10228	GCACAGCCTCGTCAAACGAA	CAAGGACCGTGAGCGTTGAG
CotAD_01886	ATGCCATTGGAGGAAGTGCTAACC	ACCGTTCACCATTAGGTCACCTTC
CotAD_62726	CAGTCTTGGCCTCAACATCAATGC	TGCAGCACTGCTTGTGGAGTTAC
CotAD_23018	CCCGGCATAGCTTGGCAATA	TGCCCTATCCAACCACTCTGT
CotAD_21782	GGCGGCCAACCTGTTCAACTC	CCGGCAAGTCCAGTGACATTCC
CotAD_21208	AATGTTATCGGCTCTCACCAAGGC	AACTGGAACTGCCATCACCACTTC
CotAD_36479	AGACAATCCTTAGGTTCGCCAAGC	TAGGAGGCGATGTCAACGAGGAG
CotAD_23542	TTCGTCGAATTGTCCGCATCCG	CGAAGAACGACTCGTCGCATGG
CotAD_69249	ACCGACATACCTCCGACACCTG	AACCAGCCATCCACCTAAGAATGC
CotAD_37676	GTAGCCACACTGTTTCGGCA	TTCGTGCTCCTTCGATTCGC
CotAD_11602	CACATCGAGCGGCATGGACTG	TTCTGGCACAATGGAACCTTCTGG
CotAD_16406	GAGAACACAAGCCGGAGATAGCG	CGTCAGCAGCCTTCCTAGACATTC
CotAD_66679	ACCATCACCACCACCAGTATCTCC	GGTGTCGTGTCATCTGTAGTTGGC
CotAD_24232	AGAACAGGGTCGGGGTTTGTGTCAC	CCAGCACTGGTGCTTCCATTGCC
CotAD_22544	TGCGGTGGGATGATGTTGCTG	GCGTTTTGCCAGTTCCAGGAG
